# NMDA-driven dendritic modulation enables multitask representation learning in hierarchical sensory processing pathways

**DOI:** 10.1073/pnas.2300558120

**Published:** 2023-07-31

**Authors:** Willem A. M. Wybo, Matthias C. Tsai, Viet Anh Khoa Tran, Bernd Illing, Jakob Jordan, Abigail Morrison, Walter Senn

**Affiliations:** ^a^Institute of Neuroscience and Medicine (INM-6) and Institute for Advanced Simulation (IAS-6) and JARA-Institute Brain Structure–Function Relationships (INM-10), Jülich Research Center, DE-52428 Jülich, Germany; ^b^Department of Physiology, University of Bern, CH-3012 Bern, Switzerland; ^c^Department of Computer Science - 3, Faculty 1, RWTH Aachen University, DE-52074 Aachen, Germany; ^d^Laboratory of Computational Neuroscience, École Polytechnique Fédérale de Lausanne, CH-1015 Lausanne, Switzerland

**Keywords:** dendritic computation, contextual adaptation, multitask learning, contrastive learning, self-supervised learning

## Abstract

While sensory representations in the brain depend on context, it remains unclear how such modulations are implemented at the biophysical level, and how processing layers further in the hierarchy can extract useful features for each possible contextual state. Here, we demonstrate that dendritic N-Methyl-D-Aspartate spikes can, within physiological constraints, implement contextual modulation of feedforward processing. Such neuron-specific modulations exploit prior knowledge, encoded in stable feedforward weights, to achieve transfer learning across contexts. In a network of biophysically realistic neuron models with context-independent feedforward weights, we show that modulatory inputs to dendritic branches can solve linearly nonseparable learning problems with a Hebbian, error-modulated learning rule. We also demonstrate that local prediction of whether representations originate either from different inputs, or from different contextual modulations of the same input, results in representation learning of hierarchical feedforward weights across processing layers that accommodate a multitude of contexts.

## Significance

In deep learning, the standard approach to accommodate changing task demands is to train new output layers on top of a common trunk network, and, if needed, to relearn synapses throughout the whole network. However, the brain appears to take a radically different strategy, as neurons in all processing layers are modulated by contextual information. We show that context-dependent dendritic afferents can powerfully modulate the neuronal output and that this modulation dynamically reshapes network function to solve new tasks, without adapting any feedforward synapses. We furthermore show that these dendritic modulations could underlie self-supervised learning of deep networks, without relying on the backpropagation of errors across the layers of the network.

**Fig. 1.**
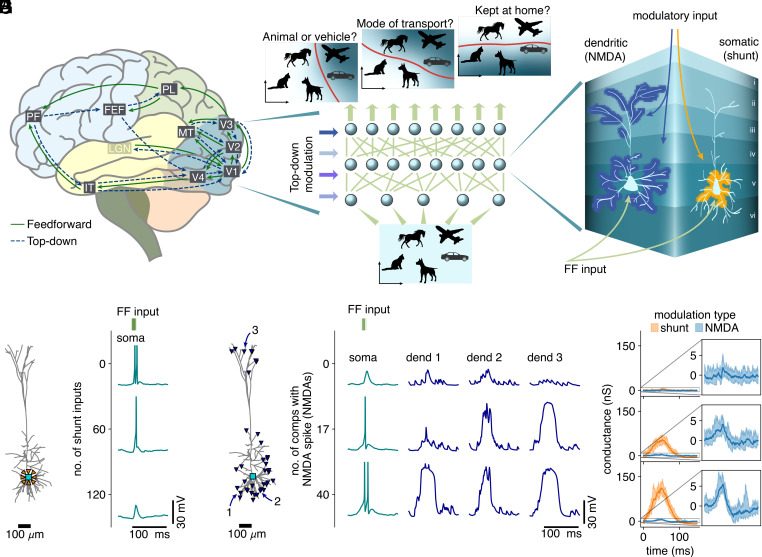
Contextual modulation of neurons in sensory processing pathways. (*A*) Top-down connections from prefrontal and motor areas relay high-level information to early sensory processing neurons [adapted from Gilbert et al. (11), LGN: lateral geniculate nucleus of the thalamus, V1-4: visual area 1-4, MT: medial temporal area, IT: inferior temporal cortex, PL: parietal lobe, FEF: frontal eye field, PF: prefrontal cortex]. (*B*) We hypothesize that high-level information from prefrontal and motor areas modulates the activity of early sensory neurons, enhancing response properties of neurons with task-relevant receptive fields. These modulations induce a task-dependent functional remapping of sensory processing pathways built on fixed, task-agnostic feedforward connectivity. (*C*) At the biophysical level, we investigate two plausible candidate mechanisms that could implement quasi-tonic neuron-specific modulations: somatic shunting inhibition and dendritic NMDA spikes. (*D*) L5 PC model configuration to investigate somatic shunting: feedforward and shunting (orange) inputs target the somatic compartment (teal). (*E*) The somatic response to identical feedforward inputs (*Top*, green, Gaussian burst of 175 inputs), for three modulation levels resulting in zero, one, or two output spikes. (*F*) L5 PC model configuration to investigate dendritic modulation: modulatory inputs target up to 38 (also corrected in *SI Appendix*, methods) dendritic compartments (blue, locations 1, 2, and 3 are the dendritic sites plotted in *G*), whereas feedforward inputs target the somatic compartment (teal). (*G*) Somatic responses (*Left*, teal) to identical feedforward inputs (*Top*, green, Gaussian burst of 40 inputs), for three different levels of dendritic modulation (*Right*, blue) resulting in zero, one, or two output spikes. (*H*) Comparison of effective conductance changes, as measured at the soma, between shunt and NMDA modulation for the three modulation levels shown in (*E* and *G*).

Sensory processing in the brain is commonly thought of as proceeding through an increasingly abstract and invariant hierarchy of representations (1, 2). According to this view, neurons have a fixed tuning to specific stimuli: In early sensory areas, neurons identify basic features such as lines, gratings (3), or simple auditory waveforms (4), while neurons further in the processing stream are selective to faces (5, 6), speakers (7), or words (8). Artificial neurons in feedforward network models also exhibit such receptive field properties, and similarity between responses in these networks and in sensory brain regions lends support to this view of sensory processing (9, 10). However, the activity of sensory neurons is not driven purely by bottom-up inputs but is also modulated by internal mental states (11). These modulating inputs, relayed by top-down connections from various cortical areas (Fig. 1*A*), communicate high-level information about behavioral context (12–14), task demands (15–17), expectations (18–20), motor commands (19, 21, 22), and memory (23, 24).

While it is attractive to assume that such top-down connections to sensory areas adapt feedforward processing to the many contexts that may occur in natural environments, the computational utility of modulating neurons at all levels in the processing stream remains poorly understood. Such modulations induce a dependence on the contextual state in sensory representations at any given processing layer. Consequently, the next processing layer in the hierarchy has to be connected in such a way that it can extract useful features, not only for each possible sensory input but also for each possible contextual state. Most artificial neural network approaches that seek to implement multitask learning avoid this complication by defining separate output networks for each task, on top of a common trunk that generates a context-independent representation of the inputs (25, 26). Nevertheless, the pervasiveness of contextual modulation in sensory processing indicates that this adaptation is an important component of cortical computation, and reshapes the functional mapping of sensory processing pathways (Fig. 1*B*) (27). While some authors have explored modulations to early processing layers (28–31), their networks were trained through error backpropagation in a purely supervised fashion. Unsupervised, representation-based learning is considered more biologically plausible (32–34), but has not been applied to context-modulated representations.

Biophysically, the way in which modulations to sensory neurons are implemented remains unknown. A probable constraint is that contextual modulations have a longer time-scale than rapid feedforward processing, where volleys of action potentials propagate rapidly through the processing hierarchy (35, 36), their trajectories modulated by the contextual inputs. With durations of 50 to 100 ms, dendritic nonlinearities convert branch-local correlated inputs into sustained somatic depolarizations that outlast somatic action potentials by up to two orders of magnitude (37). A major driver of such dendritic spikes is the N-Methyl-D-Aspartate (NMDA) receptor present at excitatory synapses to cortical pyramidal cells (PCs) (37, 38), which furthermore has been associated with the integration of signals originating from within the brain itself (39). While dendritic NMDA spikes thus appear a suitable candidate to modulate the neuronal output according to self-generated task context (31), they have not been shown to do so in network computations with biologically realistic neuron models.

Here, we study the modulation of feedforward processing in networks of biophysically realistic neurons. By assessing effective membrane conductance changes, we find that NMDA spikes can modulate the neuronal input–output (IO) relation in a manner compatible with physiological evidence. We then study the computational features of neuron-specific modulations in abstract feedforward network models and show that these modulations allow networks without task-specific readout components to solve multiple tasks. We find that feedforward weights that extract useful information from modulated layers can indeed be learned because multitask performance increases with network depth. This in turn allows the network to learn new tasks by adapting solely the modulating synapses, and inspired us to ask whether unsupervised learning principles exist for feedforward weights that support multitask learning through neuron-specific modulations. We then show that context-modulated representations promote self-supervised learning across a hierarchy of processing layers, by providing a form of data augmentation for contrastive learning that allows deeper processing layers to extract general, high-level features, without the need for error backpropagation across layers. Thus, instead of being a complication, such modulations could constitute an integral feature of cortical learning. Finally, while the contextual modulations in abstract models are trained through gradient descent on a classification loss, we show that our approach translates to biologically realistic spiking models equipped with a Hebbian, error-modulated learning rule for the contextual synapses.

## Results

### Biophysical Implementation of Neuron-Specific Modulations.

As NMDA spikes can convert branch-local correlated inputs into sustained depolarizations at the soma, they constitute a plausible candidate mechanism for implementing contextual adaptation. However, other candidate mechanisms may be plausible as well. A sustained increase in input rate of a specific group of synapses could implement a similar modulation, but would rely on a precise network mechanism to generate such firing rates. Dendritic Ca^2+^-spikes also implement sustained depolarizations, and could in principle have a similar effect on the somatic output as NMDA spikes (40). Finally, both *γ*-aminobutyric acid A and B (GABA_A_ & GABA_B_) receptors could exert influence on the neuronal IO relation through regulation of dendritic spikes (41–44). Here, we assessed whether contextual modulations would rather be implemented by dendritic or somatic afferents (Fig. 1*C*), and we did so in a biophysically realistic layer 5 (L5) PC model (45). We compared the proposed primary mechanism for dendritic modulation—NMDA spikes—with a possible mechanism for somatic modulation—shunting inhibition by fast-spiking interneurons that target the perisomatic region (46).

Conceptually, we think of the type of feedforward processing studied here as the first wave of spikes that propagates through the sensory hierarchy, e.g., following the emergence of a new feature in the visual field. Response latencies in higher cortical areas, such as in the prefrontal or the inferior temporal cortex, are 130 to 150 ms (35, 36). For this reason, we examined conditions where, for identical feedforward input, the modulatory afferents change the number of somatic outputs between zero and two spikes, as subsequent spikes would be unlikely to drive this short-latency component of the sensory response. Similarly, the feedforward inputs themselves were implemented as short Gaussian bursts, with a width of 6 ms. Since the somatic modulatory mechanism is inhibitory, we tuned the number of feedforward inputs per burst so that two output spikes were emitted without modulatory input (175 feedforward inputs), and increased the number of shunt inputs until all output spikes were prevented (Fig. 1 *D* and *E*). Conversely, as the dendritic mechanism is excitatory, we tuned the number of feedforward inputs per burst so that no output spikes were emitted without modulatory input (40 feedforward inputs), and increased the number of inputs eliciting dendritic NMDA-spikes until two output spikes were emitted (Fig. 1 *F* and *G*).

An experimentally testable measure that distinguishes between the candidate mechanisms is the change in effective conductance of the neuron. The time course of this conductance can be measured in voltage clamp by repeating the same input pattern at different holding potentials and is given by the slope of the current-voltage relationship at all time points (47). Experimental studies estimated effective conductance changes of 1 to 10 nS (47, 48). In the case of somatic modulations through shunting inhibition, our simulations showed that the effective conductance change required to modulate the output firing from two to zero spikes is between 100 and 150 nS, values far outside the experimentally measured range (Fig. 1*H*). Conversely, the effective conductance change for modulating output firing from zero to two spikes with dendritic NMDA-spikes is between 1 and 10 nS. This demonstrates that dendritic NMDA-spikes are a biologically plausible candidate to implement neuron-specific modulations (Fig. 1*H*), on which we will focus in the remainder of this work.

### Neuron-Specific Modulations as Bias and/or Gain Changes.

Conceptually, neuron-specific modulations can be thought of as changing the slope and/or threshold of the neuronal IO relationship. In abstract neuron models of the form

**Fig. 2.**
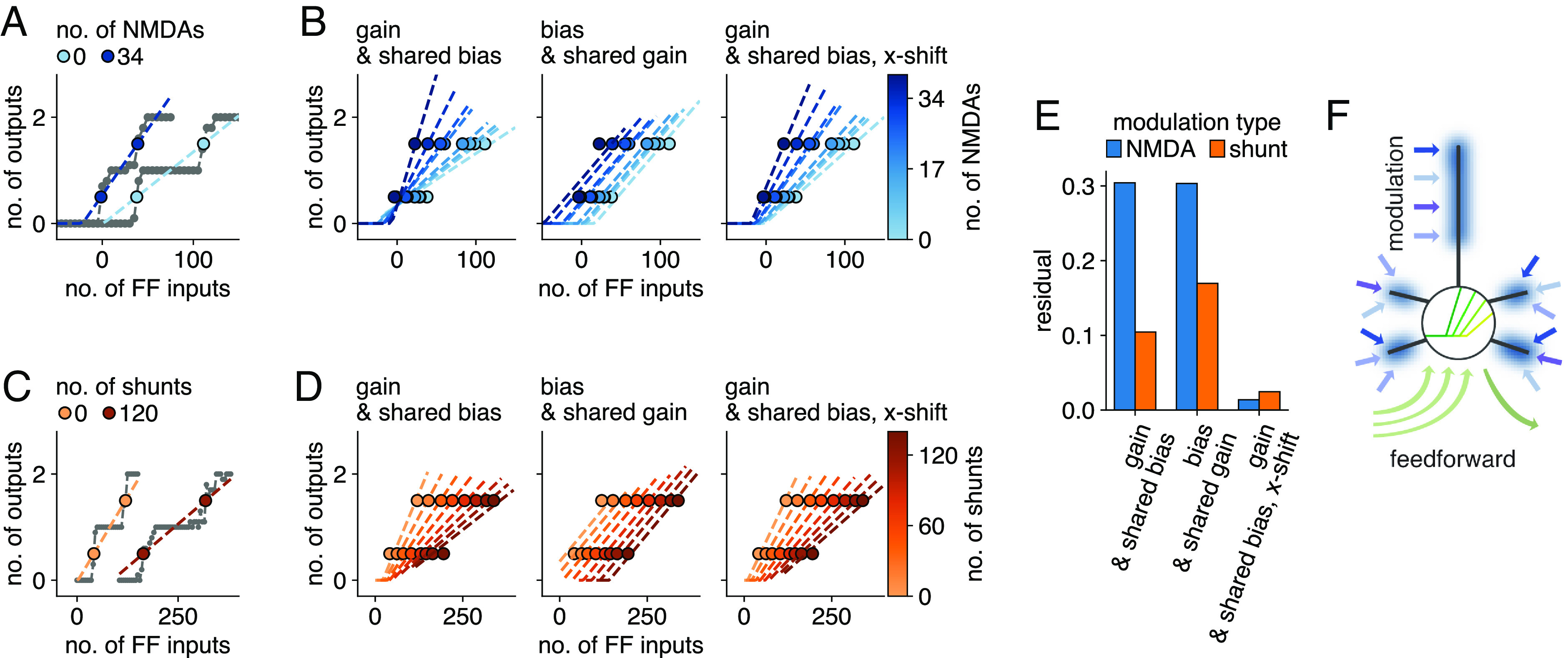
Conceptualizing neuron-specific modulations in abstract neuron models. (*A*) Number of output spikes, averaged over ten trials, for two example levels of modulation. Modulation levels are determined by the number of compartments with NMDA spikes (no. of NMDAs—light blue is without NMDA spikes, dark blue with 34 compartments with NMDA spikes). The *x*-axis shows the number of excitatory feedforward inputs (> 0) or inhibitory feedforward inputs (< 0). The thresholds (blue) where the number of emitted spikes increases are taken as the points where the linear interpolation crosses the mid-point between discrete values. We use these thresholds as fit points for the ReLU characterizing the neuronal IO relationship (dashed lines show these fits, performed here for each modulation level separately for illustrative purposes). (*B*) ReLU fits to obtained threshold values (as explained in *A*) for eight modulation levels with a curve-specific gain and shared bias (*Left*), a curve-specific bias and shared gain (*Middle*), and a curve-specific gain, shared bias, and additionally a shared x-shift (*Right*). We fitted all these modulation levels together by minimizing the sum-of-squares error. (*C* and *D*) Same as (*A* and *B*), but for modulation through somatic shunting. (*E*) Residual sum-of-squares error for the eight modulation levels, computed for the three cases shown in *B* (blue) and *D* (orange). (*F*) Proposed conceptual model of a neuron participating in sensory feedforward processing: perisomatic feedforward inputs (green) are modulated by dendritic subunits (blue), resulting in a concerted change of slope and threshold of the neuronal IO curve.

[1] $$\begin{array}{c} y=\sigma (g\;w^{T}\;x+b), \end{array}$$

this can be implemented through modulations of gain *g* and bias *b*, with *g* primarily affecting the slope and *b* exclusively affecting the threshold. Here, *y* represents the neuronal activation, *σ* the activation function, **w** the feedforward weight vector, and **x** the feedforward input vector. Note that although *y* typically stands for the average neuronal firing rate, here, we interpret it rather as the average number of somatic output spikes in response to a short burst of feedforward inputs. In this case, the ReLU activation function *σ*(*x*)=max(*x*, 0) is a reasonable choice (Fig. 2*A* and *C*).

We maintained the same input configuration to the L5 PC model as before (Fig. 1*F*) and constructed IO curves for different levels of modulation by varying the number of feedforward inputs. We then modeled the effect of modulation on the IO dependency either as gain or bias adaptation. To fit these curves, we retained the thresholds—computed as the points where the interpolation line crossed the mid-point between discrete values—as the fit points (Fig. 2*A*). We then fitted all obtained curves, either with a curve-specific gain and shared bias (Fig. 2*B*, *Left*) or with a curve-specific bias and shared gain (Fig. 2*B*, *Middle*), by minimizing the sum-of-squares error for all modulation levels together (*SI Appendix*, *Methods*). We found that the accuracy of both bias-modulated and gain-modulated fits, as quantified by the residual sum-of-squares error (Fig. 2*E*), could be improved substantially by introducing a constant *x*_shift_ parameter

[2] $$\begin{array}{c} \begin{array}{cc} y & =\sigma (g\;\left(w^{T}\;x-x_{\;\text{shift}}\right)+b)\\ & =\sigma (g\;w^{T}\;x+\left(b-g\;x_{\;\text{shift}}\right)), \end{array} \end{array}$$

resulting in concerted additive and multiplicative modulation by gain changes. This fit produced the most accurate representation of the modulatory effect (Fig. 2*B*, *Right* and *E*). Together, these considerations suggest a conceptual picture of sensory neurons where perisomatic feed-forward inputs are modulated by top-down inputs impinging onto dendritic subunits (Fig. 2*F*). These modulatory inputs increase IO slope and decrease IO threshold. For completeness, we note that somatic modulation through shunting inhibition was better fitted by pure gain modulation than bias modulation (Fig. 2*C*–*E*, configuration as in Fig. 1*D*), in agreement with prior work (46), and that introducing a constant x-shift parameter also decreased the residual markedly.

### Multitask Learning with Task-Dependent Modulations to Individual Neurons.

**Fig. 3.**
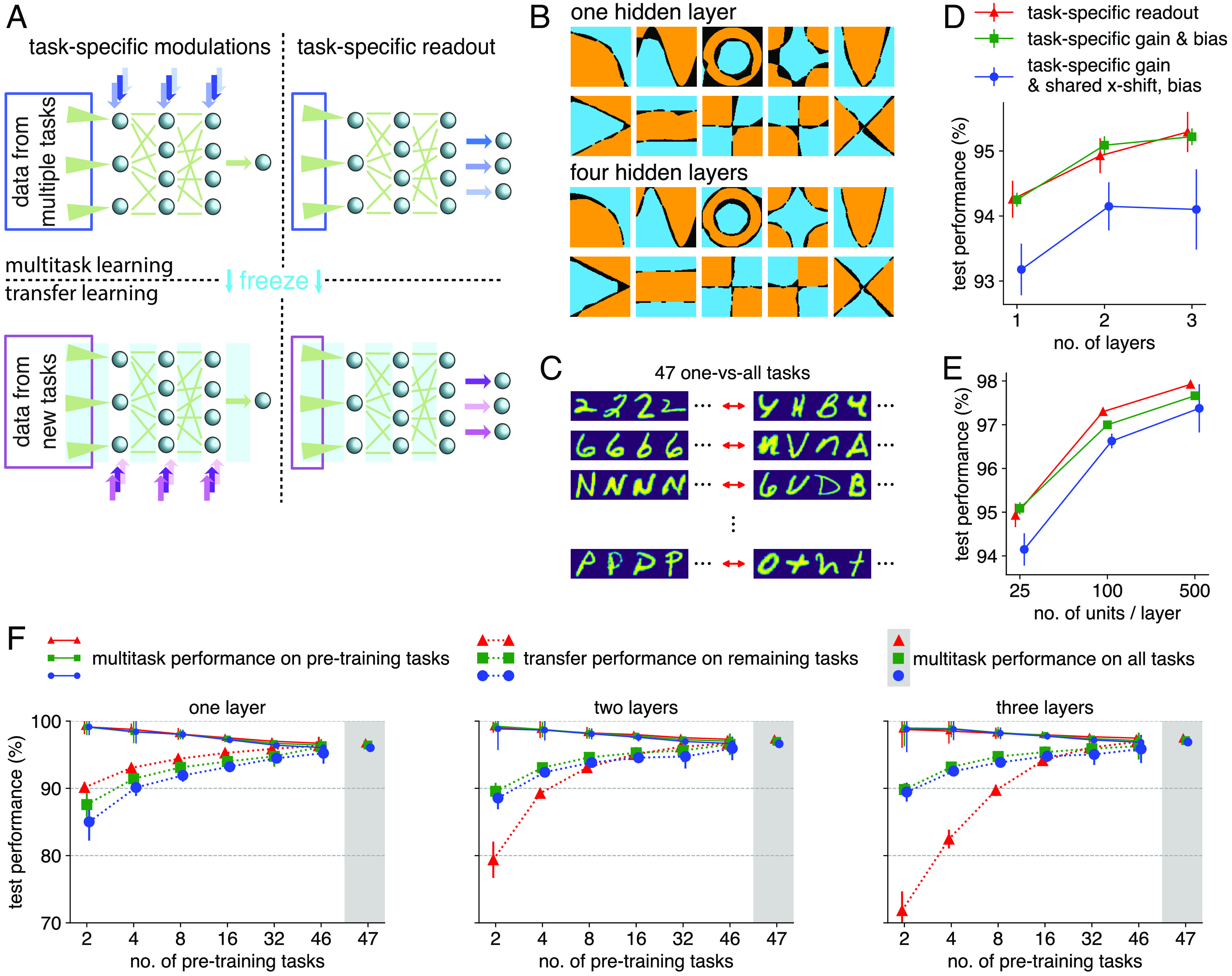
Multitask and transfer learning in feedforward networks. (*A*) The networks solve multiple tasks with a shared set of feedforward weights (green), either through different sets of neuron-specific modulations (*Left*) or different readout neurons (*Right*). In multitask learning (*Top*), shared parameters (feedforward weights, green) are trained on multiple tasks in concert with task-specific parameters for each of those tasks. In transfer learning, shared parameters are frozen (bright blue), and new task-specific parameters are learned for the new tasks (purple). (*B*) 10 (out of 48, *SI Appendix*, Fig. S1*A*) exemplars of a two-dimensional classification multitask dataset solved with neuron-specific modulations. Correctly classified samples are plotted in blue and orange, while incorrectly classified samples are plotted in black. The network architecture contains one hidden layer (*Left*) or four hidden layers (*Right*) with 50 neurons per layer, followed by a single output unit. (*C*) EMNIST is converted to a multitask learning problem by defining a one-vs-all classification task for every class in the original dataset. (*D*) Performance of networks with a task-specific readout and no neuron-specific modulations (red, triangle), independently learnable task-specific gain and bias (green, square) and task-specific gain together with a shared x-shift and bias (blue, circle) as a function of the number of hidden layers (25 neurons per layer). Performance is measured by averaging over all tasks, and by additionally averaging over five initialization seeds (error bars show standard deviation of task performance across seeds, averaged over all tasks). (*E*) Same as in *D* but for a varying number of units per layer in networks with two hidden layers. (*F*) Transfer vs. multitask learning performance. Networks are pretrained on various subsets of tasks with the normal multitask approach, yielding test performances on the pretraining tasks (full line, small marker size). Shared parameters are then frozen and only the task-specific parameters are trained on the remaining, unseen tasks. This transfer learning approach yields test performances for the transfer tasks (averages and standard deviations computed across 128 seeds, dotted lines, large marker size). Each hidden layer consists of 100 units, and multitask performances of the equivalent architecture on the full set of tasks are shown on the *Right*. Colors and markers for the different algorithms as in (*D*).

In feedforward neural network architectures, implementing task switching by providing neuron-specific modulations to the neurons in the hidden layers is a departure from the standard approach, in which task-specific output units are trained on top of a shared trunk network (Fig. 3*A*) (25, 26). We therefore first assessed whether multitask learning in this manner is even computationally feasible, and learned task-specific gains to the individual neurons in feedforward networks together with feedforward weights, x-shifts, and biases that were shared across tasks. All parameters were optimized through supervised error backpropagation.

To demonstrate that neuron-specific modulations can successfully change the functional mapping of feedforward processing pathways, we trained networks with one or four hidden layers to solve 48 binary classification tasks on two-dimensional inputs. These networks, each with a single set of feedforward weights, but task- and neuron-specific gains, solved all 48 tasks, demonstrating that such modulations achieve multitask learning (Fig. 3*B* and *SI Appendix*, Fig. S1*A*). The deeper network was more accurate (less black area in Fig. 3*B* and *SI Appendix*, Fig. S1*A*), indicating that multilayer architectures with neuron-specific modulations are computationally useful.

To more thoroughly test neuron-specific modulations on a dataset that is both sufficiently rich in tasks and sufficiently simple to subsequently combine with biophysical models, we converted the EMNIST dataset (49) into a multitask learning problem (multitask EMNIST) by defining a one-vs-all classification task for every class in the original dataset (47 tasks, Fig. 3*C*). We found that implementing neuron-specific modulations through independent gain and bias changes achieved the same performance as a task-specific readout, and that combined gain and bias changes through a constant x-shift resulted in a slightly reduced performance (Fig. 3 *D* and *E*). Qualitatively, the same behavior was observed for both investigated forms of neuron-specific modulations: performance increased with network depth (Fig. 3*D*), and performance increased strongly with layer size (Fig. 3*E*). Hyperparameters, such as learning rates, were optimized for each method and architecture separately (*SI Appendix*, Fig. S1*B*). Note that we have also implemented other neuron-specific modulations (*SI Appendix*, Fig. S1*C*), but the minute differences between modulation types could not be decoupled fully from choices such as network architecture, task design, and training method.

In the brain, mounting evidence suggests that top-down inputs dynamically select salient features from a stable feedforward connectivity (24). Our framework can replicate this strategy by making use of prior knowledge, encoded in the learned feedforward weights, and learn previously unseen tasks purely with neuron-specific modulations. By dividing our dataset in a subset of tasks to pretrain shared parameters and the remaining subset of tasks to be learned only with task-specific parameters, we were able to transform our multitask problem in a transfer learning problem. For networks with one hidden layer, we found that all approaches achieve similar transfer learning. For networks with more than one hidden layer, our approach transferred much better to the remaining tasks than networks with task-specific readouts (Fig. 3*F*). Presuming that with more hidden layers, networks become increasingly adept at filtering out task-irrelevant information, we hypothesized that task-specific readouts for new tasks have no access to information that was not relevant for the original tasks. Conversely, neuron-specific modulations to early layers could recover such information, leading to improved transfer learning.

### Unsupervised Weight Matrices for Networks with Neuron-Specific Modulations.

So far our supervised results have demonstrated that a network with a single set of feedforward weights, and contextual modulations to individual neurons, can solve many tasks. However, much of the learning in the brain is thought to proceed in an unsupervised fashion (50, 51). While unsupervised learning has been studied thoroughly in combination with a supervised readout on the hidden representation (33, 52), it has yet to be combined with neuron-specific modulations. We therefore investigated how to find unsupervised feedforward weight matrices that facilitate the construction of task-specific decision boundaries through supervised learning of the neuronal gains.

**Fig. 4.**
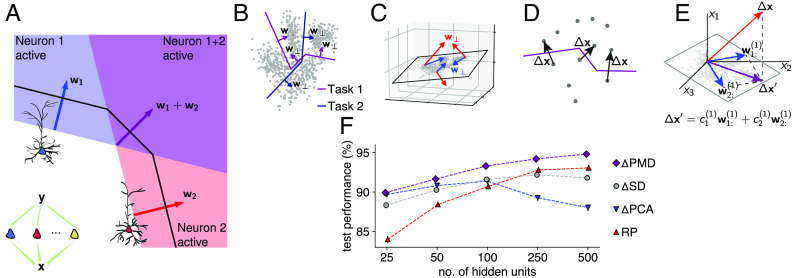
Properties of feedforward weights for networks to perform well in concert with neuron-specific modulations. (*A*) The normal vectors associated with segments of the decision boundary capture the local features that the network uses to make decisions about data sample identities (note that with ReLU units, the decision boundary consists of linear sections). In any network architecture (here, a single hidden layer, *Inset*), these normal vectors are weighted sums of the input weight vectors to the first layer neurons. (*B*) To learn a multitude of tasks with the same feedforward weights, task-relevant normal vectors to the decision boundaries must be constructible with the network. (*C*) Normal vectors to decision boundaries must be constrained to the subspace of the input data. Normal vectors outside this subspace have components orthogonal to it, which do not add useful directions for decision boundaries. (*D*) Generic decision boundaries can be constructed by concatenating segments with normal vectors close to difference vectors between close, but differently classified data points. (*E*) Combining considerations *A*–*D*, we investigate loss functions that minimize the difference between, on the one hand, difference vectors between data points and, on the other hand, their projections on the subspace spanned by the weight vectors to the first layer neurons. (*F*) Performance on multitask EMNIST (averaged over five initialization seeds) as a function of layer size for networks with neuron-specific modulations and feedforward weights given by *Δ*PMD (purple diamonds), *Δ*SD (gray circles), *Δ*PCA (blue triangles), and random projections (RP, red triangles).

To explain our approach, we note that the decision of any given neuron in the feedforward pathway to become active represents a decision boundary on the sensory input space. Locally, this boundary is characterized by its normal vector (*SI Appendix*, *Methods*), which captures the input features that the neuron uses to make a decision about whether to become active, and is always a linear combination of the input weight vectors to the network (Fig. 4*A*). A necessary condition to be able to construct a given decision boundary is that its normal vectors can all be constructed with the feedforward weight matrices (Fig. 4*B*). Our rationale, thus, is that neuron-specific modulations select a concatenation of decision boundary segments with constructible normal vectors that optimally approximates the desired decision boundary. By consequence, input weight vectors are preferentially constrained to the subspace of the data, so that all constructible normal vectors also lie within this subspace (Fig. 4*C*). When there is no a priori information on the decision boundaries that might be drawn through the data, a reasonable heuristic for the constructible normal vectors is that they approximate the set of difference vectors between data samples. In turn, decision boundaries can be seen as a concatenation of segments with normal vectors that are close to difference vectors between nearby, but differently classified data samples (Fig. 4*D*). Consequently, by aligning the set of constructible normal vectors of the network to the set of difference vectors between data samples, we ensure that constructible normal vectors lie within the data subspace, and that they constitute useful putative decision boundary directions.

To achieve such alignment, we minimized the residual min_**c**_ ∥*Δ***x**^*T*^−**c**^*T*^*W*∥_2_ between any given difference *Δ***x** and its optimal reconstruction as a linear combination of input weight vectors (the rows of the input weight matrix *W*, Fig. 4*E*) with respect to *W* for a representative set of differences

[3] $$\underset{C,W}{\arg\min}{\left\|\Delta X-CW\right\|}_{2}.$$

In this reconstruction loss, *ΔX* is a matrix with as rows the difference vectors and *C* the matrix with as rows the optimal coefficients **c**. We note that supervised training of weights and gains also decreased the residual of the reconstruction loss, and reached a much lower value in case of multitask learning than for task-specific networks (*SI Appendix*, Fig. S2). We minimized Eq. **3** in three different ways (*SI Appendix*, Table S1 and Fig. S3*A*). First, in the matrix *W* that optimizes Eq. **3** without regularizer or constraint (for lower hidden layer dimensionality *k* than input dimensionality *n*), the rows are given by the principal components of *ΔX* (*Δ*PCA), and this problem can be solved in a biologically plausible manner through Hebbian learning rules (32). Second, to encourage alignment between input weight vectors and difference vectors, we asked that any given *Δ***x** can be expressed with few weight vectors. We achieved this by adding an L1-regularization term *λ*∥*C*∥_1_ to Eq. **3**. Thus, Eq. **3** became the canonical sparse dictionary learning problem (*Δ*SD) (53, 54), which can also be solved by neural networks with biologically plausible Hebbian learning rules (33). Finally, we encouraged input weight vectors to capture local pixel correlations. We achieved this by placing an L1 constraint on ∥**w**_*j*:_∥_1_ ≤ *ϵ* on the rows of *W*, next to an L1 constraint ∥**c**_:*j*_∥_1_ ≤ *δ* for the columns of *C*. This doubly constrained minimization is known as the penalized matrix decomposition (*Δ*PMD) (55).

We then froze these feedforward weight matrices *W* and embedded them in a network architecture with a single hidden layer of gain-modulated neurons, with shared x-shift and bias. The hidden neurons targeted a single gain-modulated output unit through identical feedforward weights, and task-specific gains were trained in a supervised fashion. We found that solving Eq. **3** for differences between data samples instead of the data samples themselves generally resulted in a performance increase when combined with neuron-specific modulations to solve multitask EMNIST (*SI Appendix*, Fig. S3*B*). Assessing the relationship between input dimensionality (*n* = 784) and dimensionality of the hidden layer (number of hidden neurons *k*), we found that *Δ*PCA performed well for low numbers of hidden neurons, but that task performance saturated quickly and decreased for *k* ≥ 100 (Fig. 4*F*, blue). This result is in agreement with our theoretical considerations: When the effective dimensionality of the data is reached, further orthogonal components do not contribute usefully to the decision boundary, as they lie outside of the subspace of the input data. In contrast, using random projections (RP) in *W* by sampling from a Gaussian distribution resulted in performances that increase strongly with *k* (Fig. 4*F*, red). This can be understood by considering that with increasing numbers of random vectors, it becomes more likely that their linear combinations can approximate difference vectors between data points. Finally, we found that *Δ*PMD reached the highest performances for all *k* (Fig. 4*F*, purple). These weight vectors being sparse likely facilitates learning performant sets of neuron-specific modulations, as up- or down-regulating a specific hidden neuron influences only a localized area of the input space. By consequence, neurons with receptive fields in other areas of the input space do not need readjustment, whereas neurons with nonlocal receptive fields would need to be readjusted. In these optimizations, the shared x-shift and bias, as well as the learning rate, were optimized through an evolutionary algorithm for each configuration separately (*SI Appendix*, Fig. S3*C*).

### Task-Modulated Contrastive Learning for Stacking Processing Layers.

Sensory processing in the brain is thought to proceed in a hierarchical manner through a number of processing layers (9, 10). Deep artificial networks also implement hierarchical processing through a stack of layers, the learning of which is orchestrated by error backpropagation (56, 57). Nevertheless, the question of whether this algorithm could plausibly be implemented in the brain is still a matter of debate (58), in contrast to representation learning approaches such as PCA (32) or SD (33), which have biologically plausible implementations. These representation learning approaches, however, do not extract higher-order features when stacked in a deep network (52). Furthermore, by introducing neuron-specific modulations to the hidden processing layers, the representation learning problem becomes even more complex, as now the hidden representations depend on task modulation.

**Fig. 5.**
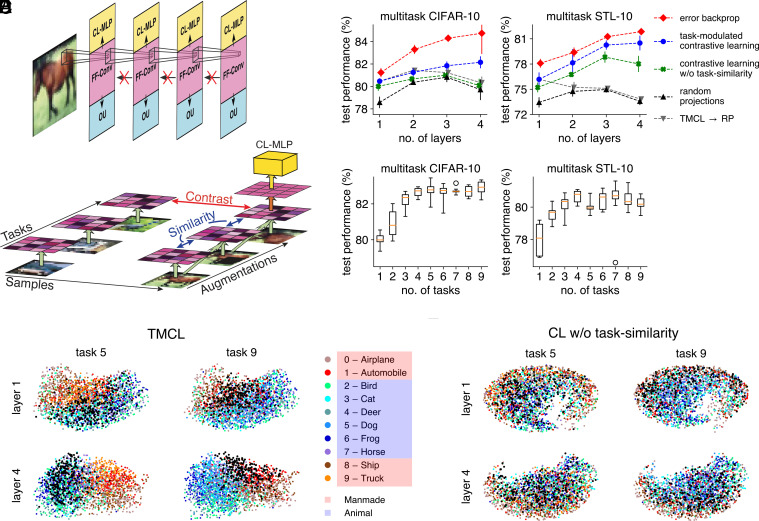
Hierarchical stacking of task-modulated convolutional layers. (*A*) We train a stack of gain-modulated convolutional layers on multitask CIFAR-10 and multitask STL-10 using a contrastive learning (CL) objective. Each layer consists of a CL multilayer perceptron (CL-MLP, yellow) to implement the CL objective, a set of convolutional feedforward weights (purple), and an output unit (blue) to learn the task-specific gains. In this task-modulated contrastive learning (TMCL) paradigm, no error gradients flow back between layers. (*B*) To learn the convolutional feedforward weights to the next layer (orange arrow), the CL-MLP maximizes contrast between representations in the last learned layer that originate from different data samples, and similarity between representation that originate from augmentations (occlusions, scalings, rotations, and combinations thereof) of the same data sample to which, additionally, different task modulations are applied (green arrows represent the feedforward pathway up until the last learned layer). (*C*) Performances averaged over five initialization seeds on multitask CIFAR-10 (*Left*) and multitask STL-10 (*Right*) for the gain-modulated networks (with shared x-shift), with filters trained by: error backpropagation (red), TMCL (blue), contrastive learning without similarity maximization across task modulations (CL w/o task-similarity, green), given by random projections (RP, black), or RP stacked on top of a TMCL layer (gray). (*D*) Multitask CIFAR-10 (*Left*) and multitask STL-10 (*Right*) performances of TMCL for networks with four layers, where during the TMCL phase similarity was maximized only over a subset of tasks, and the x-axis value denotes the no. of tasks in the subset (ten random but distinct subsets where evaluated for each number of tasks). Median performance, orange; box denotes [Q1, Q3] over the ten subsets, max. whiskers extent is five times the interquartile range (i.e., Q3–Q1), circles denote values outside of the max. whisker extent. Note that no. of tasks = 1 is the same as CL w/o task-similarity. (*E*) UMAP projections of the hidden, task-modulated representations from CIFAR-10 for the TMCL-trained network. Color code as in the legend, except that the class to be recognized is black (“dog” for task 5 and “truck” for task 9). (*F*) Same as *E*, but for the CL-trained network without task-similarity.

We propose a representation learning algorithm that does not rely on error backpropagation between layers, and where the task dependence of the hidden representations is an integral feature that improves generalization. As we have shown above, sparse feedforward connectivity is beneficial in concert with neuron-specific modulations. We therefore applied our algorithm to a convolutional architecture (Fig. 5*A*), which by design features localized receptive fields adapted for visual processing (59). Our representation learning approach takes inspiration from a successful contrastive learning (CL) algorithm (60). In this algorithm, augmentations (e.g., occlusions, rotations, scalings, and combinations thereof) are applied to the input data and the convolutional feedforward network creates hidden representations thereof. A multilayer perceptron (CL-MLP)—applied to these hidden representations—is trained in concert with the convolutional feedforward weights to maximize similarity between representations if they originate from augmentations of the same input sample; conversely to maximize contrast if they originate from different input samples. In the original formulation (60), the CL-MLP is applied once at the end of the feedforward pathway, and weight changes are orchestrated across layers by error backpropagation of the CL loss. Here, we constructed our networks layer by layer by applying this algorithm in a layer-wise fashion. Hence, a local CL-MLP minimized the CL loss to learn the feedforward weights between the previous and the current layer, and no error gradients propagated across layers (Fig. 5*A*, see *SI Appendix*, *Methods* for details). After this CL phase, we learned task-specific gains for the hidden neurons in the current layer through a task-independent output unit (OU) that maximized classification performance in a supervised manner (Fig. 5*A*, blue). To train feedforward weights to the next layer in the next CL phase, the task modulations learned in the previous layers were treated as additional data augmentations, across which similarity had to be maximized (Fig. 5*B*).

We tested our task-modulated contrastive learning (TMCL) algorithm on multitask CIFAR-10 (61) and multitask STL-10 (62), and found that network performance, averaged over all tasks, increased with the number of layers (Fig. 5*C*, blue). To establish a performance envelope, we tested equivalent network architectures trained in a fully supervised manner through end-to-end error backpropagation (Fig. 5*C*, red). The performance of networks with RP started at lower values and did not increase as much, or even decreased, across layers (Fig. 5*C*, black). Similarly, stacking RP layers on top of a TMCL layer did not increase performance across multiple layers (Fig. 5*C*, gray). Finally, removing similarity maximization across task representations from TMCL also abolished the performance increase with stacking (Fig. 5*C*, green). We furthermore assessed network performance while using different numbers of tasks over which to maximize similarity. For each number of tasks, we selected ten random but distinct subsets containing that amount of tasks. During the CL phase, only those tasks were different across representations originating from the same sample. We then evaluated network performance across all tasks and found that performance increased with subset size (Fig. 5*D*), indicating that maximizing similarity across many tasks improves generalization.

Finally, we visualized how the network constructs and modulates hidden representations, to investigate whether high-level information was extracted across layers. We applied the uniform manifold approximation and projection [UMAP, (63)], a nonlinear visualization method, to TMCL-generated hidden representations. In the first layer, at most a general distinction between manmade objects (red shades) and animals (blue shades) could be observed, while in the fourth layer individual classes appeared in a localized pattern (Fig. 5*E*). Such localized patterns were also found with error backpropagation (*SI Appendix*, Fig. S4*A*), but could not be distinguished for CL without task-similarity (Fig. 5*F*) or for RP (*SI Appendix*, Fig. S4*B*).

### Spiking Networks with Biophysically Realistic Dendritic Branches Learn Task Switching Online.

**Fig. 6.**
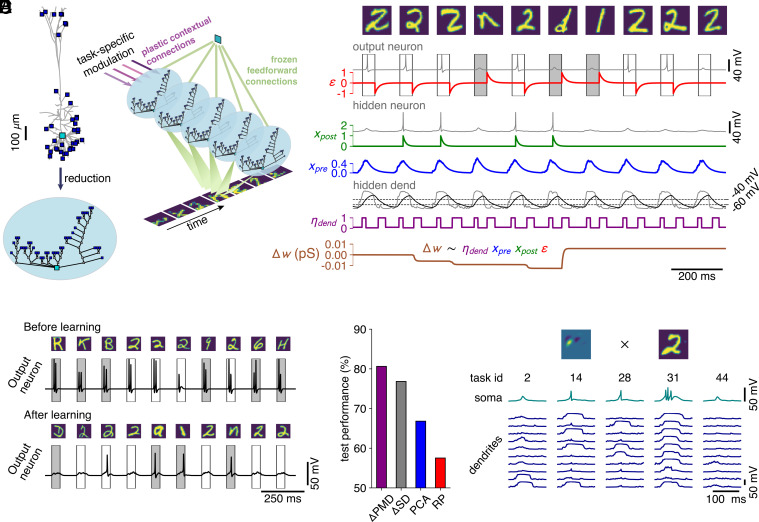
Dendritic branches learn to solve multitask EMNIST through a biologically plausible learning rule. (*A*) To simulate a feedforward network consisting of neurons with biophysically realistic dendritic subunits for a sufficiently long time, we reduce the L5 PC and synapse configuration shown in Fig. 1*F* (64). We then connect these neurons to an output neuron—implemented as a single-compartment reduction of the same model— that learns to spike in response to a random sample and to remain silent in response to a sample from the class to be recognized. (*B*) Weight changes of dendritic synapses (brown, *Bottom*) are computed as the product of a global error signal (red), a low-pass filter of the postsynaptic spikes (green), a low-pass filter of the presynaptic spikes (blue) and a voltage-dependent learning rate modulation (purple). (*C*) Voltage trace of the output neuron before learning (*Top*) and after learning (*Bottom*), for the network with the *Δ*PMD feedforward weight matrix, during an examplar one-vs-all task (not spiking in response to “two”). Note that the apparent variability in spike amplitude is due to the recording time step of 1 ms. (*D*) Performance on multitask EMNIST of the resulting model for the different feedforward weight matrices, labels as in Fig. 4*F*. (*E*) Somatic voltage (teal) and a subset of dendritic voltages (blue) in a representative hidden neuron, for the same feedforward input [i.e., the input weights (*Top*, *Left*) that scale the synaptic inputs originating from a randomly chosen data sample (*Top*, *Right*)] and five example tasks (*Left* to *Right*). Similarly to C, variability in spike amplitude is due to a recording time step of 1 ms.

We have shown in this paper that networks of ReLU neurons can learn to implement multitudes of tasks with a form of gain modulation that models the impact of dendritic NMDA-spikes on somatic output. It remains to be shown, however, that inputs to biophysically realistic dendritic branches—eliciting NMDA-spikes—can indeed modulate the spiking output of neurons in an orchestrated manner and to a sufficient degree of precision, so that the network as a whole can solve many tasks. To demonstrate this, we maintained a structure similar to the model with one hidden layer investigated previously (Fig. 4), but replaced the gain-modulated ReLU neurons with spiking models that have realistic dendritic subunits. We simplified the L5 PC model (*SI Appendix*, Fig. S5) using the method developed in our previous work (64), obtaining a model that was computationally sufficiently inexpensive to permit the network to be run over long timescales, thus allowing us to present a large amount of inputs. The output neuron was a single compartment model, obtained by only fitting the soma of the full L5 PC model, whereas the hidden layer consisted of 100 neurons, each equipped with 40 dendritic compartments where context-modulating AMPA+NMDA synapses impinged (Fig. 6*A*, blue). Feedforward weights to the somata of the hidden neurons were given by the *Δ*PMD, *Δ*SD, PCA, or RP matrices. We think of the feedforward weights to the output neuron as being unspecific, perhaps a direct connection path to a brain area trying solve tasks with low-level information. Before the specific weights in such a path are learned, the targeted brain area can already solve tasks by providing global error-feedback to dendritic contextual synapses in the early sensory area. We therefore implemented these weights as being uniform, but with some Gaussian variability (*σ*/*μ* = 0.1, see *SI Appendix*, *Methods* for the precise value for *μ*). All feedforward synapses (to the hidden neurons and to the output neuron) were static, whereas the context-modulating synapses to the dendritic compartments were subject to plasticity to learn the various tasks.

Learning at the dendritic synapses was orchestrated by an online error-weighted Hebbian plasticity rule during a continuous stream of inputs (Fig. 6*B*). Because of our choice of architecture, with similar weights from all hidden neurons to the output neuron, this learning rule approximately follows the error gradient of the classification loss (*SI Appendix*, *Methods*). For each data sample, the pixel intensities were converted into short, Gaussian bursts of spikes (width of 6 ms), with spike numbers proportional to pixel intensity. These spikes were fed into feedforward synapses, whose weights were scaled according to the matrices computed in the previous section. Conversely, the task context was encoded by a wide Gaussian burst (width of 20 ms), consisting of on average 60 spikes if the context was active and zero spikes otherwise. The first of the feedforward spikes opened a 50-ms window in which the output neuron had to either generate an output spike— in response to a random sample — or generate *no* output spike — in response to a sample from the class to be recognized. In case of erroneous firing, a global error signal (Fig. 6*B*, red) was relayed to the dendritic synapses of the hidden neurons. This error signal was then multiplied by a low-pass filter of the somatic spike output (Fig. 6*B*, green), a low-pass filter of the presynaptic spike input (Fig. 6B, blue), and a learning rate modulation (Fig. 6*B*, purple) based on a low-pass filter of the local dendritic voltage (Fig. 6*B*, black).

This network architecture solved multitask EMNIST, and tasks that are demonstrably not linearly separable, such as XOR (*SI Appendix*, Fig. S6). Initially, the output neuron fired indiscriminately but learned to spike correctly during the target intervals (Fig. 6*C* and *SI Appendix*, Fig. S6 *C* and *D*, shaded boxes). Assessing network performances averaged over all 47 tasks (Fig. 6*D*), we found that performance differences observed between alternative feedforward matrices for the artificial network architecture (Fig. 4*F*) were exacerbated, with RP performing barely better than chance level. Thus, in the noisy and imprecise spiking system, it is all the more important that the feedforward weight matrix consists of localized receptive fields, well-adapted to the input data. Our *Δ*PMD matrix achieves this for multitask EMNIST. Finally, we assessed the somatic and dendritic activity after learning in the same hidden neuron, for the same feedforward input, across different tasks (Fig. 6*E*, in the *Δ*PMD-network). We found that between zero and three output spikes were emitted, depending on the precise dendritic state. Thus, this network successfully learned multitask EMNIST by expressing a different dendritic state for each task. These learned dendritic states modulated rapid feedforward processing to solve a multitude of tasks, supporting our central hypothesis.

## Discussion

In this work, we have proposed dendritic NMDA-spikes as a mechanism for contextual adaptation, and have shown that they can modulate the neuronal output in a manner compatible with the biological constraints. The resulting neuron-specific modulations can reshape the functional mapping of sensory networks according to task context, without relying on changes to the feedforward weights. As individual NMDA-spikes in dendritic branches contribute only a small amount to the somatic depolarization, the ensemble of branches implements a graded modulation of the somatic output. In turn, this allows a Hebbian, error-modulated plasticity rule to orchestrate gradient-based learning of the dendritic synapses to a sufficient degree of accuracy, so that the network as a whole can solve many tasks. We have also shown that task modulations to hidden layers can augment sensory representations, facilitating the extraction of high-level features through contrastive learning without relying on the backpropagation of errors across processing layers.

While the component of TMCL that learns task modulations can be implemented in a biologically plausible fashion, as shown through our network model with realistic dendritic subunits, the contrastive learning step in our study relies on precise error backpropagation through the CL-MLP. However, a contrastive learning algorithm has recently been proposed in the context of predictive coding that relies solely on Hebbian learning rules (34). This algorithm shows that contrastive learning could be implemented in a self-supervised manner, by neurons connecting locally to principal feedforward cells, and using gaze information to assess whether similarity or contrast has to be maximized.

Aside from the somatic channels and NMDA receptors, the membrane of the L5 PC model was fully passive, as this was the only technically feasible way to implement simulations with multitask learning. As a consequence, the contribution of apical contextual inputs to the somatic voltage may be underestimated in our L5 PC model. By modeling the active properties of the L5 PC cell through a full complement of Ca^2+^, Na^+^, and K^+^ channels distributed in the apical dendrite (45), we nevertheless demonstrated that dendritic modulation through both apical and basal NMDA inputs into active dendrites can be captured in our general framework (*SI Appendix*, Fig. S7). Thus, Ca^2+^-spikes could provide complementary modulation by amplifying NMDA-spikes in the apical compartments. Finally, our results do not exclude GABA_A_ and GABA_B_ receptor-mediated regulation of dendritic spike generation as another contextual signal.

Our work suggests that top-down dendritic modulation can complement feedforward activity to nudge neural responses toward desired activities. If consistent across contexts, a modulation thus constitutes a target for the feedforward input, yielding a natural relation to theories of dendritic error representation (65–67). This potential combined role of dendrites for error representation and contextual modulation is corroborated by some early evidence (68).

A puzzling observation, first discovered in high-level areas (69, 70) and later also in early sensory regions (71–73), is that the participation of a neuron in the representation of a sensory stimulus changes over time. This representational drift raises questions about the framework of classical representation learning, and about how stable perception can be achieved (74, 75). As we show, changes to the sensory representation could help in extracting high-level information in further processing layers. The drift itself could be a manifestation of changes in the internal mental state—encoded on dendritic trees and thus invisible in most imaging experiments.

Feedforward processing needs to be rapid, for instance, to initiate evasive action when a threat is identified, while contextual modulation likely proceeds on a slower time scale, for instance to bias the feedforward pathway toward detection of relevant threats given an environment. We have linked this difference in time scales to the underlying biophysical processes: the short duration of somatic spikes (1 to 5 ms) and by extension the whole feedforward pathway (100 to 150 ms) (35, 36) in comparison to the duration of dendritic spikes [50 to 100 ms, or possibly longer (37, 76)]. These temporal scales match the frequency bands associated with feedforward processing (gamma, 60 to 80 Hz) and top-down processing (alpha–beta, 10 to 20 Hz) observed across a large range of tasks and stimuli (77–82).

Another interesting aspect of dendritic NMDA-spikes is that they function as branch-local, semi-independent feature detectors (83). In the brain, the contextual signal to a neuron is likely a rich combination of cross-modal information, recurrent information about the recent past, and top-down signals about high-level goals, behavioral state, and environment characteristics. Spatially segregated feature detection allows neurons to robustly infer context from all these different signals by preventing spurious activations by random subsets of inputs (84). Local recurrent connections target basal and proximal apical dendrites (85, 86), and may relay information about the recent past as a context for the present. Axons carrying top-down signals primarily target L1, indicating that the apical tree is an important locus for the integration of contextual information (87). To a lesser extent, these axons also target L5 and L6 (88, 89), indicating that contextual information provided by local recurrent circuitry may still be augmented by top-down modulations.

Taken together, our work reframes feedforward processing in the brain as a fundamentally adaptable process, steered dynamically by contextual inputs that modify the dendritic state. Our theory matches environmental constraints to the underlying biophysical layout, and may help to explain diverse observations, such as the frequency bands associated with feedforward and top-down processing, and the apparent instability of sensory representations.

## Materials and Methods

The L5 PC model (45) was equipped with membrane parameters to reproduce the amplitudes of glutamate-uncaging evoked NMDA-spikes in L5 PC dendrites and somata. The model was then targeted by excitatory synapses to the dendritic compartments featuring both AMPA and NMDA receptors, while current-based feedforward synapses impinged on the soma. Simulations were performed using NEAT (64) and NEURON (90). With a custom PyTorch (91) data sampler, we then ensured that the data for multitask learning was balanced across tasks and task-classes. The IO relation of an abstract network layer with task-specific gain & shared x-shift & bias, was described by

[4] $$\begin{array}{c} y=\sigma \left(g_{t}^{\left(l\right)}⊙\left(W^{\left(l\right)}\;x-x_{\;\text{shift}}^{\left(l\right)}\right)+b^{\left(l\right)}\right), \end{array}$$

with as shared parameters the weight matrix *W*^(*l*)^, the x-shift $x_{\;\text{shift}}^{\left(l\right)}$ and the bias **b**^(*l*)^, and as task-specific parameters the gains **g**_*t*_^(*l*)^. *σ* was the ReLU activation and l=1,⋯,L the layer index. We then employed five learning schemes for the parameters: i) multitask learning through supervised error backpropagation on all parameters (Fig. 3), ii) transfer learning by freezing the shared parameters (pretrained in the multitask setup) and supervised error backpropagation on the task-specific parameters (Fig. 3), iii) unsupervised learning of *W*^(1)^, combined with supervised learning of **g**_*t*_^(1)^ (Fig. 4, in these simulation $x_{\;\text{shift}}^{\left(1\right)}$ and *b*^(1)^ were scalar and treated as metaparameters, *SI Appendix*, Fig. S3*C*). iv) In the convolutional setup (Fig. 5), the analogue of *W*^(*l*)^ is the set of convolutional filters, which were trained through layer-local task-modulated contrastive learning, while gains **g**_*t*_^(*l*)^ of each layer were trained through layer-local supervised learning. v) In the biophysical network setup (Fig. 6), each entry of *W*^(1)^—pretrained through (iii)—was proportional to the weight of a single feedforward synapse. Feedforward synapses then remained frozen, while the AMPA+NMDA synapses in the dendritic compartments were learned in an online fashion through a Hebbian, error-modulated learning rule. Full simulation details and mathematical derivations can be found in *SI Appendix*, *Methods*.

## Supplementary Material

Appendix 01 (PDF)

## Data Availability

Computer code and intermediate simulation data have been deposited in Zenodo (https://doi.org/10.5281/zenodo.7870103) (92).
