# Supplementary material for: NMDA-driven dendritic modulation enables multitask representation learning in hierarchical sensory processing pathways
Source: Proc Natl Acad Sci U S A. 2023 Jul 31;120(32):e2300558120. doi: 10.1073/pnas.2300558120 (PMC10410730; doi:10.1073/pnas.2300558120)
Supplement: Supplementary file 1 — Appendix 01 (PDF) [file pnas.2300558120.sapp.pdf]

PNAS

Supporting Information for

NMDA-driven dendritic modulation enables multitask representation learning in hierarchical sensory processing pathways

W.A.M. Wybo, M.C. Tsai, V.A.K. Tran, B. Illing, J. Jordan, A. Morrison, and W. Senn

Willem A. M. Wybo.
E-mail: willem.a.m.wybo@gmail.com

This PDF file includes:

Supporting text
Figs. S1 to S7
Tables S1 to S5
SI References

Supporting Information Text

Methods

Biophysical modelling. The morphology and ion channels for the L5 PC model were taken from Hay et al. (1) and implemented in the NEURON simulator (2). In Figs 1, 2, and 6, we only retained the most important somatic Na^+ and K^+ channels (NaTa and Kv3.1) and used a passive dendritic membrane, with physiological parameters that were proposed by Major et al. to reproduce the amplitudes of glutamate-uncaging evoked NMDA-spikes in L5 PC dendrites and somata (3), combined with a spine correction as in Rhodes et al. (4). For Fig S7, we used the parameters for the fully active L5 PC as provided by template 1 in Hay et al. (1).

Contextual and background synapses were conductance based, either containing AMPA+NMDA (excitatory) or GABA (inhibitory) receptors. AMPA and GABA receptors were implemented as the product of a double exponential conductance profile (5) g with a driving force:

$$i_{\text{syn}} = g\,(e_r - v), \quad \text{with} \quad g = w\,n(\tau_r, \tau_d)\,\left(e^{-t/\tau_d} - e^{-t/\tau_r}\right). \qquad [1]$$

Here, e_r is the synaptic reversal potential, τ_r and τ_d are the synaptic rise and decay time constants, and n a normalization constants that depends on τ_r and τ_d and normalizes the peak of the conductance window to the synaptic weight w. AMPA rise and decay times were $\tau_r = 0.2$ ms, $\tau_d = 3$ ms and AMPA reversal potential was $e_r = 0$ mV. For GABA, we set $\tau_r = 0.2$ ms, $\tau_d = 10$ ms and $e_r = -80$ mV. N-methyl-D-aspartate (NMDA) currents (6) were implemented as:

$$i_{\text{syn}} = g\,\sigma(v)\,(e_r - v) \qquad [2]$$

with $\tau_r = 0.2$ ms, $\tau_d = 43$ ms, and $e_r = 0$ mV, while $\sigma(v)$ – the channel's magnesium block – had the form (7):

$$\sigma(v) = \frac{1}{1 + 0.3\,e^{-0.1\,v}}. \qquad [3]$$

The weight w of a synapse signifies the maximum value of its conductance profile. For an AMPA+NMDA synapse, the weight is the maximal value of the AMPA conductance profile, and the maximal value of the NMDA profile is twice that of the AMPA window (NMDA ratio of 2). Feedforward synapses were current based, with a dual exponential current profile with $\tau_r = 0.2$ ms and $\tau_d = 3$ ms. The excitatory synaptic weight corresponded to the maximal value of the current profile. For inhibitory feedforward synapses, the weight was negative and corresponded to the minimum value.

For dendritic modulation, we identified 20 dendritic compartments suited for semi-independent NMDA-spike generation (e.g. avoiding compartments on sister branches, Fig 1F) and equipped each of these compartments with an AMPA+NMDA synapse. Both the feedforward and shunt inputs targeted the somatic compartment. The 20 dendritic compartments as well as the soma were also equipped with an AMPA and GABA background synapse. All parameters for the simulations are shown in Table S1.

	Dendritic modulation	Somatic modulation
Weight exc (inh) feedforward synapses	0.06 (-0.06) nA	0.06 (-0.06) nA
Feedforward inputs burst width	6 ms	6 ms
No. of AMPA+NMDA inputs	60	
High AMPA+NMDA weight	0.08 nS	
Low AMPA+NMDA weight	0.001 nS	
No. of comps with NMDA spike	{0, 6, 11, 17, 23, 29, 34, 40}	
Shunt weight		5 nS
No. of shunt inputs		{0, 20, 40, 60, 80, 100, 120}
Contextual inputs burst width	20 ms	40 ms
Soma comp AMPA (GABA) background rate	100 (200) Hz	100 (200) Hz
Dend comps AMPA (GABA) background rate	60 (120) Hz	60 (120) Hz
Weight AMPA (GABA) background synapses	1 (1) nS	1 (1) nS

Table S1. Simulation-specific parameters to investigate somatic and dendritic modulation.

Note that for dendritic modulation, we set the number of compartments with NMDA-spikes n_c by targetting n_c compartments with an AMPA+NMDA synapse of high weight and $20 - n_c$ compartments with an AMPA+NMDA synapse of low weight, and delivered the same amount of input spikes in each case. For somatic modulation, the weight of the shunt synapses remained the same, but the number of input spikes was modified. Note that the burst width for somatic modulation was enlarged to account for the shorter time-scale of the GABA conductance window compared to the NMDA conductance window.

To measure the membrane conductance change induced by the contextual inputs (Fig 1H), we followed Haider et al.(8) and measured the time-dependent electrode current in voltage clamp ($i(t; v_h)$, with v_h the holding potential) for two different holding potentials: $v_{h1} = -80$ mV and $v_{h2} = 0$ mV. Following the Ohmic relationship $g(t)\,(v_h - v_0) = i_h(t; v_h)$, with v_0 the equilibrium potential, we found the membrane conductance as

$$g(t) = \frac{i(t; v_{h1}) - i(t; v_{h2})}{v_{h1} - v_{h2}}. \qquad [4]$$

To visualize $g(t)$ as in Fig 1H, we subtracted the pre-stimulus baseline and plotted the min-max envelope obtained over 10 trial runs.

IO curve parameter fitting. To obtain the IO curves (Fig 2), we changed the number of feedforward inputs in each burst (weights as in Table S1), and either had only excitatory inputs (no. of feedforward inputs > 0) or inhibitory inputs (no. of feedforward inputs < 0). For each number of inputs, we presented ten independently sampled bursts featuring that number of inputs, and measured the average number of output spikes generated in response.

To fit the IO curves, we used a linear least squares fit ($\text{argmin}_{\mathbf{u}} \|A\,\mathbf{u} - \mathbf{y}\|_2$) both for the shared gain and shared bias cases, but constructed the feature matrix A and parameter vector $\mathbf{u}$ differently. On the domain where $y > 0$, the IO curve is

$$\begin{aligned} &y = g_m\, n_{\text{ff}} + b \quad \text{for gain modulation} \\ &\text{and} \\ &y = g\, n_{\text{ff}} + b_m \quad \text{for bias modulation,} \end{aligned} \tag{5}$$

with n_{ff} the number of feedforward inputs. For M modulation levels, we have parameter vectors $\mathbf{u} = (g_1, g_2, \ldots, g_M, b)$ for gain modulation and $\mathbf{u} = (g, b_1, b_2, \ldots, b_M)$ for bias modulation, and corresponding feature matrices

$$A = \begin{bmatrix} n_{\text{ff}1}|_{m=1} & 0 & \ldots & 0 & 1 \\ n_{\text{ff}2}|_{m=1} & 0 & \ldots & 0 & 1 \\ 0 & n_{\text{ff}1}|_{m=2} & \ldots & 0 & 1 \\ 0 & n_{\text{ff}2}|_{m=2} & \ldots & 0 & 1 \\ \vdots & \vdots & \ddots & \vdots & \vdots \\ 0 & 0 & \ldots & n_{\text{ff}1}|_{m=M} & 1 \\ 0 & 0 & \ldots & n_{\text{ff}2}|_{m=M} & 1 \end{bmatrix} \tag{6}$$

for gain modulation and

$$A = \begin{bmatrix} n_{\text{ff}1}|_{m=1} & 1 & 0 & \ldots & 0 \\ n_{\text{ff}2}|_{m=1} & 1 & 0 & \ldots & 0 \\ n_{\text{ff}1}|_{m=2} & 0 & 1 & \ldots & 0 \\ n_{\text{ff}2}|_{m=2} & 0 & 1 & \ldots & 0 \\ \vdots & \vdots & \vdots & \ddots & \vdots \\ n_{\text{ff}1}|_{m=M} & 0 & 0 & \ldots & 1 \\ n_{\text{ff}2}|_{m=M} & 0 & 0 & \ldots & 1 \end{bmatrix} \tag{7}$$

for bias modulation, with $n_{\text{ff}1}|_m$ corresponding to the number of feedforward inputs where the output transitioned from 0 to 1 spikes and $n_{\text{ff}2}|_m$ from 1 to 2 spikes, given a modulation level m. The output vector $\mathbf{y} = (0.5, 1.5, 0.5, 1.5, \ldots, 0.5, 1.5)$ was identical in both cases. To extend gain modulation with a shared x-shift, we modified the entries in the gain modulation feature matrix A from $n_{\text{ff}i}|_m$ to $n_{\text{ff}i}|_m - x_{\text{shift}}$ $(i = 1, 2,\ m = 1, \ldots, M)$ and minimized the fit residual $\min_{\mathbf{u}} \|A(x_{\text{shift}})\,\mathbf{u} - \mathbf{y}\|_2$ over the x_{shift} values.

Supervised learning in the fully connected networks. The fully connected network architectures with neuron-specific modulations (Fig 3) consisted of a fixed number of layers of equal size, the final layer connected to a single output unit. The neuron-specific modulations were implemented in an abstract fashion, as task-specific parameters (detailed mathematical description of each model in Table S2), although it is straightforward to extend the network architecture to implement the modulations as synaptic inputs. The networks were trained through end-to-end error backpropagation. For the networks with task-specific readouts, the hidden units had unit gain (fixed) and a trained bias shared across tasks, and as many output units as there were tasks. The hidden layer neurons had ReLU transfer functions and the output unit a $\tanh(\cdot)$ transfer function. The networks were implemented in PyTorch (9), trained on batches of size 1 000 using Adam (10). We computed the mean squared error between target and network output as loss. The targets were defined as either -1 or $+1$, and a custom sampler assured batches consisted of an equal number of samples from each task and task-class. The training was interrupted with an early stopping criterion with a patience of 5 epochs on the validation performance evaluated after each epoch on a validation set of 47932 samples, or after 50 epochs, whichever came first. Independent learning rates for shared and task-specific parameters were optimized for each form of multitask leaning separately with an iterative grid search (Fig S1C). Performances were measured by averaging over all tasks, and by additionally averaging over twenty initialization seeds (error bars show standard deviation of task-performance across seeds, averaged over all tasks). In the case of transfer learning, each of the twenty pseudo-random seeds led to an independently drawn random subset of tasks (with fixed subset size) that was used to train all model parameters with gradient descent using the multitask learning approach described above. For each seed, all the available tasks that were not used in pre-training were then learned by adapting only the task-dependent parameters of the model (see Table S2). This transfer learning phase also employed early stopping on a validation set to stop training and evaluated test performance on a testing set of data samples unused during any phase of training.

Model type	Mathematical formulation	Task-dependent parameters
task-specific readout	$\mathbf{y}^{(l)} = \sigma\left(W_t^{(l)}\,\mathbf{x} + \mathbf{b}_t^{(l)}\right)$ *	$\{W_t^{(l)}, \mathbf{b}_t^{(l)} \mid l = L\}$
task-specific gain & bias	$\mathbf{y}^{(l)} = \sigma\left(\mathbf{g}_t^{(l)} \odot (W^{(l)}\,\mathbf{x}) + \mathbf{b}_t^{(l)}\right)$	$\{\mathbf{g}_t^{(l)}, \mathbf{b}_t^{(l)} \mid 1 \leq l \leq L\}$
task-specific gain & shared x-shift, bias	$\mathbf{y}^{(l)} = \sigma\left(\mathbf{g}_t^{(l)} \odot (W^{(l)}\,\mathbf{x} - \mathbf{x}_{\text{shift}}^{(l)}) + \mathbf{b}^{(l)}\right)$	$\{\mathbf{g}_t^{(l)} \mid 1 \leq l \leq L\}$
task-specific gain & shared x-shift	$\mathbf{y}^{(l)} = \sigma\left(\mathbf{g}_t^{(l)} \odot (W^{(l)}\,\mathbf{x} - \mathbf{x}_{\text{shift}}^{(l)})\right)$	$\{\mathbf{g}^{(l)} \mid 1 \leq l \leq L\}$
task-specific gain & shared bias	$\mathbf{y}^{(l)} = \sigma\left(\mathbf{g}_t^{(l)} \odot (W^{(l)}\,\mathbf{x}) + \mathbf{b}^{(l)}\right)$	$\{\mathbf{g}_t^{(l)} \mid 1 \leq l \leq L\}$
task-specific bias	$\mathbf{y}^{(l)} = \sigma\left(W^{(l)}\,\mathbf{x} + \mathbf{b}_t^{(l)}\right)$	$\{\mathbf{b}_t^{(l)}\ 1 \leq l \leq L\}$
task-specific network	$\mathbf{y}^{(l)} = \sigma\left(W_t^{(l)}\,\mathbf{x} + \mathbf{b}_t^{(l)}\right)$	$\{W_t^{(l)}, \mathbf{b}_t^{(l)} \mid 1 \leq l \leq L\}$

Table S2. Comparison of the task-dependenent parameters for the different mulitask and transfer learning approaches.

The mathematical formulations describe the output vector $\mathbf{y}^{(l)}$ for neurons in layer l with the input vector $\mathbf{x}$ (corresponding either to the output of the previous layer or the input data vector) for a feedforward network of L layers solving a task t. Note: $\odot$ denotes the Hadamard product (element-wise multiplication) and σ describes the element-wise transfer function mapping a vector of pre-activations in layer (l) to an output vector. * In the case of task-specific readouts $W_t^{(l)}$ and $\mathbf{b}_t^{(l)}$ are only task-dependent in the last layer ($l = L$)

Decision boundary normal vectors. To explain our approach, we consider the pre-activation $a : \mathbb{R}^n \to \mathbb{R} : \mathbf{x} \to a(\mathbf{x})$ of a neuron in a feedforward network as a function of the sensory input $\mathbf{x}$ (the activation y being given by $y(\mathbf{x}) = \sigma(a(\mathbf{x}))$). Biophysically, this quantity most closely corresponds with the somatic voltage under Na^+-channel blockage; it is the aggregate of all inputs and when it crosses a threshold, the neuron would emit a spike if the non-linear activation function (the Na^+-channels) were applied. The neuron may be active ($a(\mathbf{x}) > 0$) or inactive ($a(\mathbf{x}) < 0$), and its decision boundary on the input domain is given by the set $\text{D} = \{\mathbf{x}_\text{D} \in \mathbb{R}^n \mid a(\mathbf{x}_\text{D}) = 0\}$. In a small enough region around a point $\mathbf{x}_\text{D} \in \text{D}$, $a(\mathbf{x})$ can be approximated as being linear:

$$a(\mathbf{x}) \simeq \mathbf{w}_\perp^T(\mathbf{x} - \mathbf{x}_\text{D}), \tag{8}$$

and $\mathbf{w}_\perp := \frac{\text{d}a(\mathbf{x})}{\text{d}\mathbf{x}}\Big|_{x=x_\text{D}}$ is the local normal vector of the decision boundary. This normal vector is always a linear sum of the input weight vectors $\mathbf{w}_j$ to the first layer neurons ($j = 1, \ldots, k$, with k the number of neurons in the first layer), and is perpendicular to the local decision boundary (see methods), thus capturing the local input features that a uses to make a decision about whether to become active close to $\mathbf{x}_\text{D}$ (Fig 4A).

First, we note that for input $\mathbf{x} \in \text{D}$ on the decision boundary D close enough to $\mathbf{x}_\text{D}$, $a(x) = 0$, and thus, following Eq. (8):

$$\mathbf{w}_\perp^T\,(\mathbf{x} - \mathbf{x}_\text{D}) \approx 0. \tag{9}$$

By consequence, $\mathbf{w}_\perp$ is perpendicular to the local decision boundary and indeed its normal vector.

Second, we compute $\mathbf{w}_\perp$ explicitly, and to that purpose write the preactivation a of a neuron in layer K of the network as:

$$\begin{aligned} a &= g^{(K)}\left(\mathbf{w}^T\,\mathbf{y}^{(K-1)} - x_\text{xshift}^{(K)}\right) + b^{(K)} \\ \mathbf{y}^{(i)} &= \sigma\left(\mathbf{g}^{(i)} \odot \left(W^{(i)}\,\mathbf{y}^{(i-1)} - \mathbf{x}_\text{xshift}^{(i)}\right) + \mathbf{b}^{(i)}\right), \qquad \text{with } i = 1, \ldots, K-1,\ \mathbf{y}^{(0)} = \mathbf{x}, \end{aligned} \tag{10}$$

with $\mathbf{y}^{(i)}$ the neural activations in layer i, $\sigma : \mathbb{R} \to \mathbb{R}$ the neural activation function applied element-wise to its inputs, $W^{(i)}$ the weight matrix from layer $i-1$ to layer i, $\mathbf{g}^{(i)}$, $\mathbf{x}_\text{xshift}^{(i-1)}$ resp. $\mathbf{b}^{(i)}$ the gains, x-shifts resp. biases in layer i, w the weight vector to the neuron and g, x_xshift resp. b its gain, x-shift and bias. $\mathbf{w}_\perp$ is then found as

$$\mathbf{w}_\perp(\mathbf{x}_\text{D}) = g^{(K)}\,\mathbf{w}^T\,\frac{\text{d}\mathbf{y}^{(K-1)}}{\text{d}\mathbf{y}^{(K-2)}}\cdots\frac{\text{d}\mathbf{y}^{(2)}}{\text{d}\mathbf{y}^{(1)}}\,\frac{\text{d}\mathbf{y}^{(1)}}{\text{d}\mathbf{x}}\Bigg|_{\mathbf{x}=\mathbf{x}_\text{D}}, \tag{11}$$

with $\text{d}\mathbf{y}^{(i)}/\text{d}\mathbf{y}^{(i-1)}$ the Jacobian matrix of $\mathbf{y}^{(i)}$ with respect to $\mathbf{y}^{(i-1)}$. This Jacobian is given by

$$\text{d}\mathbf{y}^{(i)}/\text{d}\mathbf{y}^{(i-1)} = D^{(i)}\,W^{(i)}, \quad D^{(i)} = \text{diag}\left(\left(\mathbf{g}^{(i)} \odot \sigma'\left(\mathbf{g}^{(i)} \odot \left(W^{(i)}\,\mathbf{y}^{(i-1)} - \mathbf{x}_\text{xshift}^{(i)}\right) + \mathbf{b}^{(i)}\right)\right)\right). \tag{12}$$

Thus, we find for Eq. (11):

$$\mathbf{w}_\perp(\mathbf{x}_\text{D}) = g^{(K)}\,\mathbf{w}^T\,D^{(K-1)}\,W^{(K-1)}\cdots D^{(2)}\,W^{(2)}\,D^{(1)}\,W^{(1)}. \tag{13}$$

By rearranging this matrix product, we obtain

$$\begin{aligned} \mathbf{w}_\perp(\mathbf{x}_\text{D}) &= \sum_{j=1}^{k} \underbrace{\left[g^{(K)}\,\mathbf{w}^T\,D^{(K-1)}\,W^{(K-1)}\cdots D^{(2)}\,W^{(2)}\,\mathbf{d}_{:j}^{(1)}\right]}_{c_j(\mathbf{x}_\text{D})}\,\mathbf{w}_{j:}^{(1)} \\ &= \sum_{j=1}^{k} c_j(\mathbf{x}_\text{D})\,\mathbf{w}_{j:}^{(1)}, \end{aligned} \tag{14}$$

a linear weighted sum of the inputs weight vectors (with $\mathbf{w}_{j:}^{(1)}$ the j'th row of $W^{(1)}$ and $\mathbf{d}_{:j}^{(1)}$ the j'th column of $D^{(1)}$).

Abbr.	Unsupervised loss function	Constraints	Parameter values
PCA	$\left\lVert X - C^{(1)}\, W^{(1)} \right\rVert_2$	$\left\lVert \mathbf{w}^{(1)}_{j:} \right\rVert_2 = 1$	
ΔPCA	$\left\lVert \Delta X - C^{(1)}\, W^{(1)} \right\rVert_2$	$\left\lVert \mathbf{w}^{(1)}_{j:} \right\rVert_2 = 1$	
PMD	$\left\lVert X - C^{(1)}\, W^{(1)} \right\rVert_2$	$\left\lVert \mathbf{c}^{(1)}_{:j} \right\rVert_1 \leq \delta$, $\left\lVert \mathbf{w}^{(1)}_{j:} \right\rVert_1 \leq \epsilon$, $\left\lVert \mathbf{w}^{(1)}_{j:} \right\rVert_2 = 1$	$\delta = .5\sqrt{k}$, $\epsilon = .3\sqrt{n}$
ΔPMD	$\left\lVert \Delta X - C^{(1)}\, W^{(1)} \right\rVert_2$	$\left\lVert \mathbf{c}^{(1)}_{:j} \right\rVert_1 \leq \delta$, $\left\lVert \mathbf{w}^{(1)}_{j:} \right\rVert_1 \leq \epsilon$, $\left\lVert \mathbf{w}^{(1)}_{j:} \right\rVert_2 = 1$	$\delta = .5\sqrt{k}$, $\epsilon = .3\sqrt{n}$
SD	$\left\lVert X - C^{(1)}\, W^{(1)} \right\rVert_2 + \lambda \left\lVert C^{(1)} \right\rVert_1$	$\left\lVert \mathbf{w}^{(1)}_{j:} \right\rVert_2 = 1$	$\lambda = .1$
ΔSD	$\left\lVert \Delta X - C^{(1)}\, W^{(1)} \right\rVert_2 + \lambda \left\lVert C^{(1)} \right\rVert_1$	$\left\lVert \mathbf{w}^{(1)}_{j:} \right\rVert_2 = 1$	$\lambda = .1$

Table S3. The unsupervised loss functions with their constraints and parameter values.

Without regularizer or constraint, the optimum of the reconstruction loss for $k \leq n$ is given by the singular value decomposition of X or ΔX, and the rows of $W^{(1)}$ are the principal components if X is centered (ΔX is centered by definition). With the L1 regularizer, $C^{(1)}$ is commonly referred to as the sparse code (SC) and $W^{(1)}$ as the sparse dictionary (SD).

Unsupervised learning of weights combined with supervised gain modulation. The unsupervised optimization problems for PCA, ΔPCA, SC, SD, and ΔSD were solved using Scikit-learn (11), and for PMD and ΔPMD we used a custom implementation. The networks consisted of a single hidden layer, with weights to the hidden layer neurons resulting from optimising the loss functions in Table S3 (for RP, weights were drawn from a Gaussian distribution and then normalized so that $\left\lVert \mathbf{w}^{(1)}_{j:} \right\rVert_2 = 1$). Weights to the output unit were uniform, and also normalized to have unit Euclidean norm ($\mathbf{w}^{\text{out}} = (1, \ldots, 1)/\sqrt{k}$, with k the dimensionality of the hidden layer). Activation functions, target outputs, the optimizer and the way in which performance was measured were identical to the fully supervised networks.

We optimized gains for each task separately by performing gradient descent with batches of 100 samples, and performed an evolutionary meta-parameter optimization using DEAP (12) to find optimal values for x-shift, bias, and learning rate by maximizing validation performance on a subset of 10 tasks (Fig S4C).

To construct Fig S2, we evaluated the residual $\min_C \left\lVert \Delta X - C\, W \right\rVert$ of the reconstruction loss Eq. (3) during supervised training without regularizer or constraint (i.e. the least mean squares optimum with respect to C) on matrices ΔX containing 1000 differences.

Loss gradient with respect to task gains as a Hebbian learning rule with global error modulation. Here, we show that in the network architecture described in the previous section, the error gradient with respect to the task-dependent gains can be expressed as a Hebbian learning rule modulated by a global error signal. We interpret task-dependent gains to the hidden neurons with activity y_i ($i = 1, \ldots, k$) as synaptic inputs originating from task-encoding neurons. With $g_{i,t}$ the weight of the connection to neuron i associated with task t, and $z_t \in \{0, 1\}$ the activity of the associated task-encoding neuron (1 if the task is active and 0 otherwise), the total gain is $g = \sum_t g_{i,t}\, z_t$. The structure of these networks then becomes

$$\begin{aligned} y_i &= \sigma\left((\sum_t g_{i,t}\, z_t)\, (\mathbf{w}_i^T\, \mathbf{x} - x_{\text{shift}}) + b \right), \qquad i = 1, \ldots, k \\ y_o &= \tanh\left((\sum_t g_{o,t}\, z_t)\, \frac{\sum_i y_i}{\sqrt{k}} + b_o \right) \\ \mathcal{L}_t &= -\frac{1}{2}\, (y_o - \hat{y}_{o,t})^2, \end{aligned} \qquad [15]$$

with y_o the activity of the output neuron, b_o its bias and $g_{o,t}$ the weight of the task-gain connection to the output neuron. $\hat{y}_{o,t} \in \{-1, 1\}$ is the task-dependent target value for a given input sample. Computing the gradient of the task-loss $\mathcal{L}_t$ with respect to the task-gains in the hidden layer yields

$$\frac{\mathrm{d}\mathcal{L}_t}{\mathrm{d}g_{i,t}} = \underbrace{-(y_o - \hat{y}_{o,t})\, \tanh'\left(g_{o,t}\, \frac{\sum_i y_i}{\sqrt{k}} + b_o \right) \frac{g_{o,t}}{\sqrt{k}}}_{\epsilon}\; \underbrace{\sigma'\left(g_{i,t}\, (\mathbf{w}_i^T\, \mathbf{x} - x_{\text{shift}}) + b \right)\, (\mathbf{w}_i^T\, \mathbf{x} - x_{\text{shift}})}_{post}\; \underbrace{z_t}_{pre}. \qquad [16]$$

One crucial observation here is that since the feedforward weights to the output unit are all identical, they do not modify the error signal in a neuron-specific manner. By consequence, the error signal to the hidden layer is global, i.e. identical for all hidden neurons. A second crucial observation is that while it may seem as if the *post* factor could change the sign of the gradient, if

$$\mathbf{w}_i^T\, \mathbf{x} - x_{\text{shift}} < 0, \qquad [17]$$

this is actually impossible with the ReLU activation function (which results in $\sigma' \in \{0, 1\}$), and positive gains together with negative bias, as obtained from the L5 PC model. Indeed, for the neuron to be active ($\sigma' = 1$), the inputs need to be sufficiently

strong, so that

$$g_{i,t}\left(\mathbf{w}_i^T\,\mathbf{x} - x_{\mathrm{shift}}\right) + b > 0,$$
$$\Rightarrow \mathbf{w}_i^T\,\mathbf{x} - x_{\mathrm{shift}} > -\frac{b}{g_{i,t}} > 0, \qquad [18]$$

where the second inequality holds because we have negative bias and positive gains. Thus, whenever Eq. (17) is satisfied, the neuron is inactive ($\sigma' = 0$) and $\mathbf{w}_i^T\,\mathbf{x} - x_{\mathrm{shift}}$ does not influence the update of $g_{i,t}$. We will leverage this fact in the spiking neural network, where we replace the *post* factor by a low pass filter of the somatic output spikes to obtain a learning rule that follows the approximate gradient.

Spiking network. In order to simulate the network model, which consisted of one hidden layer with 100 neurons, over sufficiently long time scales to allow significant learning, we reduced the passive L5 PC model by retaining only the dendritic compartments and the soma (Fig 6A, Fig S5), using the method proposed by Wybo et al. (13) to conserve dendro-somatic response properties. We equipped each dendritic compartment with an AMPA+NMDA synapse for each context – 47 in multitask EMNIST (Fig 6) and 14 for the boolean tasks (Fig S6) – whose weight evolved according to

$$\dot{w} = \eta_{\mathrm{dend}}\, u_{\mathrm{pre}}\, u_{\mathrm{post}}\, \epsilon \qquad [19]$$

with η_{dend} a learning rate dependent on a low-pass filter u_{dend} of the local dendritic voltage v_{dend}, u_{pre} a low pass filter of the input spikes to the contextual synapse, u_{post} a low pass filter of the output spikes and ϵ a global error signal, implemented as the low pass filter of an error pulse whose amplitude a_e was proportional to the difference between the number of generated output spikes in the 'reward window' and the expected number (i.e. 0 or 1). This 50 ms reward window opened upon the arrival of the first feedforward input spike associated with a data sample. A delta pulse with amplitude a_e was then injected into ϵ at the closure time t_e of this reward window. Summarizing the above, we had the following:

$$\begin{aligned}
\dot{u}_{\mathrm{dend}}(t) &= -\frac{u_{\mathrm{dend}}(t) - v_{\mathrm{dend}}(t)}{\tau_{\mathrm{dend}}} \\
\eta_{\mathrm{dend}}(t) &= \begin{cases} \eta_0 & \text{for } u_{\mathrm{dend}}(t) < \theta_0 \\ \eta_1 & \text{for } \theta_0 < u_{\mathrm{dend}}(t) < \theta_1 \\ \eta_2 & \text{for } \theta_1 < u_{\mathrm{dend}}(t) \end{cases} \\
\dot{u}_{\mathrm{pre}}(t) &= -\frac{u_{\mathrm{pre}}(t)}{\tau_{\mathrm{pre}}} + \sum_{s \in \mathrm{pre}} c_{\mathrm{pre}}\, \delta(t - t_s) \\
\dot{u}_{\mathrm{post}}(t) &= -\frac{u_{\mathrm{post}}(t)}{\tau_{\mathrm{post}}} + \sum_{s \in \mathrm{post}} c_{\mathrm{post}} \delta(t - t_s) \\
\dot{\epsilon}(t) &= -\frac{\epsilon(t)}{\tau_\epsilon} + \sum_{e} a_e \delta(t - t_e).
\end{aligned} \qquad [20]$$

Note that η_{dend}, u_{dend}, u_{pre} and u_{post} are all specific to the synapse, whereas ϵ is global and shared across all synapses. Model parameters are summarized in Table S4.

To ensure that the combined feedforward post-synaptic potential (PSP) varied within a reasonable dynamic range, we took the feedforwards weights derived previously (ΔPMD, ΔSD, PCA, and RP to the hidden layer, and $(1, \ldots, 1)/\sqrt{k}$ to the output neuron) and applied a scale factor

$$s_w = \frac{v_{\mathrm{range}}}{\|\mathbf{w}_{\mathrm{in}}\|_1\, z_{\mathrm{in}}\, n_{\mathrm{in}}} \qquad [21]$$

to the input weight vector $\mathbf{w}_{\mathrm{in}}$ of each neuron, with z_{in} the somatic input resistance of the L5 PC, and n_{in} the number of feedforward inputs ($n = 784$ for a neuron in the hidden layer and $k = 100$ for the output neuron in multitask EMNIST, and $n = 2$ and $k = \{4, 10\}$ for the boolean tasks). v_{range} is a parameter that fine-tunes the dynamic range of the combined feedforward PSPs. For the hidden neurons, v_{range} was set heuristically to 40 mV on multitask EMNIST and 20 mV on the boolean tasks, and for the output neuron, v_{range} was set to 300 mV on multitask EMNIST and 200 mV on the boolean tasks. Together with z_{in}, this yielded feedforward weights in nA. Note that for the output weights, we additionally added Gaussian noise to the weight vector with $\sigma = 0.1\,\mu$, and $\mu = s_w/\sqrt{k}$.

To train this system on multitask EMNIST, we presented 200 000 samples for each task (balanced across task-classes, so repetition of the same sample may occur) by converting them to Gaussian input spike bursts (the number of input spikes in a burst varied between 0 and 10 and was proportional to pixel intensity), and injecting these burst in the network at 150 ms intervals. We then froze the plasticity rule and tested performance on 500 samples for each task taken from the EMNIST test set (again balanced across task-classes). For the boolean tasks, there were either no spikes in the burst when the input was 0 or 10 spikes when the input was 1.

Plasticity rule parameter	Parameter value
τ_{dend}	25 ms
η_0, η_1, η_2	10^{-4} nS/ms, 10^{-3} nS/ms, 5×10^{-5} nS/ms*
θ_0, θ_1	-60 mV, -40 mV
$\tau_{\text{pre}}, \tau_{\text{post}}$	25 ms, 10 ms
$c_{\text{pre}}, c_{\text{post}}$	1/60, 1
τ_ϵ	15 ms
**Network parameter**	**Parameter value**
No. of feedforward inputs per burst	0-10 depending on pixel intensity
Feedforward inputs burst width	6 ms
No. of AMPA+NMDA inputs per burst	60 for active context, 0 otherwise
Max AMPA+NMDA weight	0.2 nS
Min AMPA+NMDA weight	0.01 nS
Contextual inputs burst width	20 ms
Soma comp AMPA (GABA) background rate	100 (200) Hz
Dend comps AMPA (GABA) background rate	60 (120) Hz
Weight AMPA (GABA) background synapses	1 (1) nS

Table S4. Parameters used in the network model with biophysically realistic neurons.

*For the boolean tasks, η_i $(i = 0, 1, 2)$ was multiplied by a factor 10^{-1}.

Hyperparameter	Contrastive Learning phase	Task-specific modulation learning phase
batch size	4096	8192
number of epochs	100	80 (CIFAR-10), 120 (STL-10)
learning rate	{0.1, 0.01, 0.001}	{0.1, 0.01, 0.001}
gain initialization	{Constant(1), Rademacher, Rademacher+KaimingUniform, KaimingUniform}	
checkpoint selection	lowest loss on train set	lowest loss on validation set
temperature	0.5	N/A

Table S5. Hyper-parameters used in TMCL.

Sets denote that the hyper-parameter is part of a grid search with the given values.

Task-modulated contrastive learning. For TMCL (Fig 6), we converted CIFAR-10 (14) and STL-10 (15) into multitask learning problems (multitask CIFAR-10 and multitask STL-10) as before, by defining 10 1-vs-all classification tasks, and sampled data in a balanced manner across tasks and task-classes. We kept 9000 samples as validation set for CIFAR-10 and 1350 for STL-10. Each color channel was centered, then normalized to unit variance.

We implemented a visual geometry group-like (VGG) architecture following Illing et al. (16) For multitask CIFAR-10, we trained a stack of L convolutional layers with kernel size 3, stride 1 and 64 channels, and applied batch normalization (17) to the task-modulated convolution outputs before applying the ReLU activation function. For multitask STL-10, we employed a kernel size of 7 and stride of 3 in the first layer, and an identical architecture as for multitask CIFAR-10 everywhere else. Batch normalization renders the task-independent bias b obsolete; it was not included in our simulations. By consequence, the output of a convolutional unit was

$$\text{ReLU}\left(\text{BatchNorm}(g_t\left(w * x - x_{\text{shift}}\right))\right), \tag{22}$$

where x denotes the image patch, w refers to the respective convolutional filter, $*$ denotes the convolution operation, and g_t the task-specific gain. Every second layer was succeeded by a MaxPool layer with stride 2x2.

Our approach learns a stack of convolutional layers iteratively, by adding the next layer on top of the previously learnt ones. Each iteration consists of two distinct phases: first, feedforward filters to the next layer are learned through CL, and subsequently we learn task- and neuron-specific gains for the new layer in a supervised manner.

For CL phase, we followed the SimCLR algorithm (18). We used the same image augmentations that the authors used on their CIFAR-10 experiments followed by standard score normalization according to the statistics of the original dataset. To train the filters from layer $l-1$ to layer l $(l = 1, \ldots, L)$, we first generated batches of augmented hidden representations in layer $l-1$. With $x_1, \ldots, x_N$ a batch of input samples (see Table S5 for batch size), we generated an augmented batch $\tilde{x}_1, \ldots, \tilde{x}_{2N}$ of twice the original size, with samples $\tilde{x}_k, \tilde{x}_{N+k}$ being positive pairs, i.e. generated through image augmentations from the same source sample x_k. We then propagated this augmented batch through the hierarchy to layer $l-1$, while applying task-gains from a random task to each augmented sample, to obtain a batch of task-modulated hidden representations $\tilde{y}_1, \ldots, \tilde{y}_{2N}$. Next, the convolutions with the to be learned filters from layer $l-1$ to layer l were computed and the ReLU was applied to obtain the representation that was fed into the CL-MLP. The CL-MLP consisted of a hidden layer and an output layer with dimension

64, and used ReLU activation. The similarity $s_{i,j}$ between representations z_i and z_j obtained from final layer of the CL-MLP was computed as their cosine similarity

$$s_{i,j} = \frac{z_i \cdot z_j}{\|z_i\|_2 \; \|z_j\|_2}. \qquad [23]$$

The loss was computed as

$$\mathcal{L} = \frac{1}{2N} \sum_{k=1}^{N} [\ell(\tilde{y}_k, \tilde{y}_{N+k}) + \ell(\tilde{y}_{N+k}, \tilde{y}_k)],$$

with

$$\ell(\tilde{y}_k, \tilde{y}_{N+k}) = -\log \frac{\exp(s_{k,N+k}/\tau)}{\sum_{\substack{j=1 \\ l \neq k}}^{2N} \exp(s_{k,j}/\tau)}, \qquad [24]$$

and temperature $\tau = 0.5$. The error gradient of this loss was then used to train the filters from layer $l-1$ to layer l. All parameters, including convolutional filters, for layers $< l-1$ remained frozen. For $l = 1$, layer $l-1$ is the input layer and no task-modulation could be applied, so that $\tilde{y}_k \equiv \tilde{x}_k$.

To perform the classification for all one-vs-all tasks, we reduced the height and width axes by averaging, retaining a 64-dimensional vector with the per-channel averages. Finally, an output unit with $\tanh(\cdot)$ was applied to the inner product of the 64-dimensional representation vector and a learned, task-independent weight vector $\mathbf{w}_{\text{out}}$. Task-specific gains in layer l were then trained to minimize the classification loss (same as in our fully connected architectures) at the output unit (test performance at this output unit is what is reported in Fig 5B,C), and the process was continued at layer $l+1$.

Except for the task-specific gains, all parameters (i.e. the CL-MLP parameters, convolutional filters, the x-shift and the output unit) followed the Kaiming initialization, a standard approach in the VGG literature (19). For the task-specific gains, we tested the following initialization strategies

- **Constant(1):** Initialize each entry to 1.
- **Rademacher:** Sample each entry from Uniform$\{-1, 1\}$.
- **KaimingUniform:** Each entry is sampled i.i.d. from Uniform$(-\gamma, \gamma)$ with $\gamma = 2\sqrt{\frac{3}{n_{\text{in}}}}$ and where n_{in} is the number of inputs targeting a given unit.
- **Rademacher+KaimingUniform:** Sample each entry from **Rademacher**, then add a sample from **KaimingUniform** to each entry.

The final performance numbers reported were selected from the grid search described in Table S5, according to the best accuracy on the validation set. All experiments were implemented in PyTorch (9) and training was performed using Adam (10).

We compared our TMCL algorithm with four control cases: *(i)* error backpropagation, *(ii)* CL without task-similarity, *(iii)* RP, and *(iv)* stacking RP layers on top of a TMCL-trained first layer. For *(i)*, we trained both filters, x-shifts, and task-gains across all layers on the supervised classification loss. For *(ii)*, we applied the *same* task-gains to both augmented data samples in a CL pair. For *(iii)*, filters remained unchanged from their initialization, while task-gains were trained in a supervised manner. Finally, for *(iv)*, we trained the initial layer with plain TMCL and then applied the same procedure as in *(iii)*. All control experiments used the applicable parts of the grid search as outlined in Table S5. For Fig 5D, we split the tasks into two disjoint subsets, one with $T \in \{1, \ldots, 10\}$ tasks that were different across CL-pairs, and one with $10 - T$ tasks that were the same. We then sampled a random task for the first pair element, and, when the task was in the first subset, sampled another random tasks from the same subset for the second pair element. When the task was in the other subset, we applied the same task to the second pair element.

Computing systems. The simulations for Fig 1 & 2 where performed on an Apple M1 Max Macbook Pro with 32 GB of RAM. Simulations in Fig 3 where performed on the HPC cluster of the Insel Data Science Center. Simulations for Fig 6 were performed on the JURECA supercomputer at Forschungszentrum Jülich (where each compute node is equipped with 128 AMD EPYC 7742 cores at 2.25 GHz, and 512 GB DDR4 RAM at 3200 MHz). Simulations for Fig 5 were performed on accelerated computed nodes on JURECA, which are additionally equipped with four Nvidia A100 GPUs.

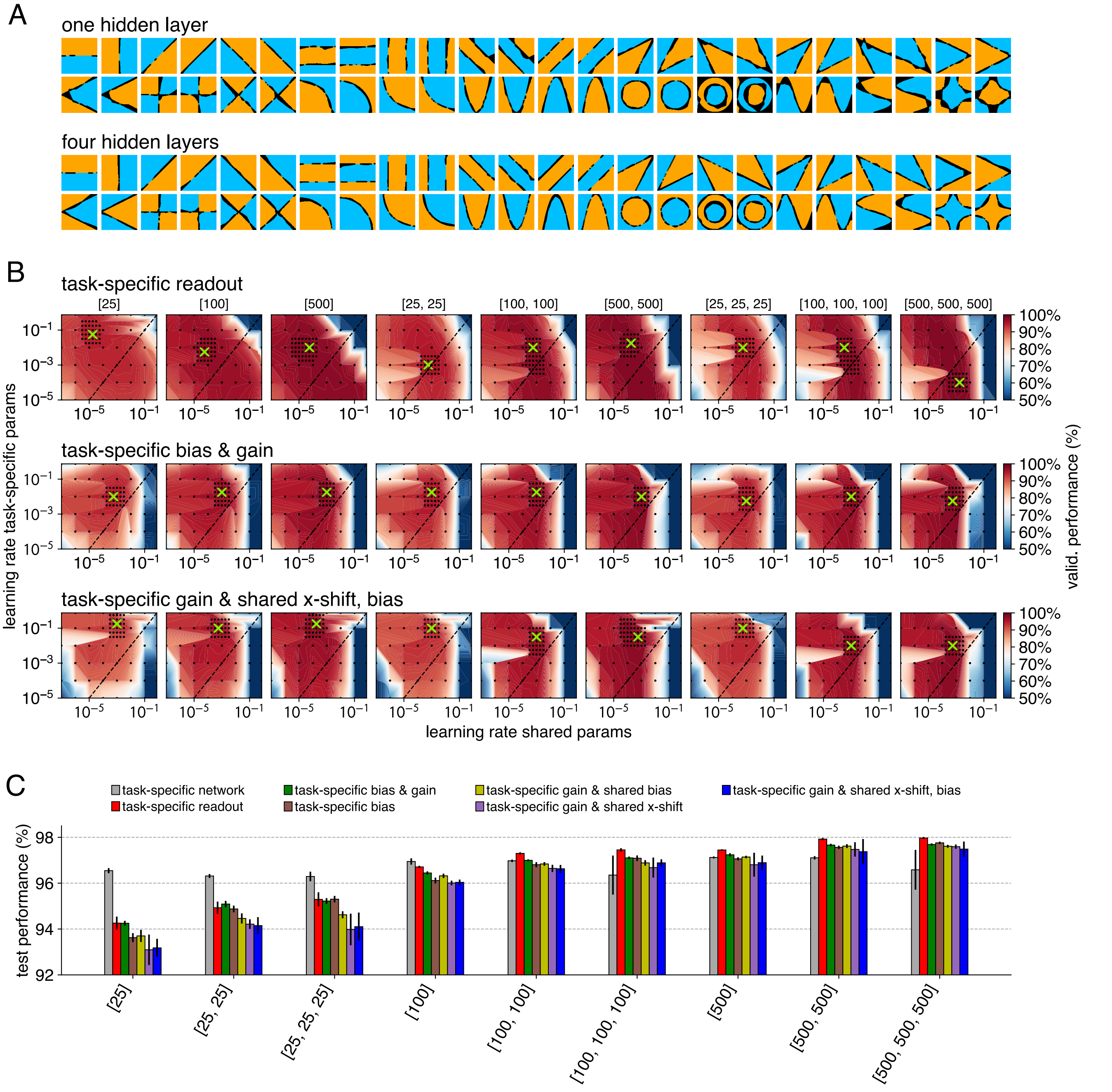

Fig. S1. Supplementary results for the networks trained in a fully supervised fashion. **A:** Full set of binary classification tasks (48), solved with a network with a single hidden layer (top) and four hidden layers (bottom) through adaptation of the task-specific gains. Each hidden layer consisted of 50 units. Feedforward weights, x-shifts and biases were shared across tasks. **B:** Results of learning rate scans for the three multitask learning approaches shown in Fig 3, for all network architectures (top labels denote the number of hidden units in each layer). For neuron-specific modulations (middle and bottom rows) the optimal (green cross) modulation learning rate (y-axis) was generally larger than the weight learning rate (x-axis). **C:** Performances on multitask EMNIST of task-specific networks (no parameters shared across tasks, grey), task-specific readouts (red) and the various possible neuron-specific modulations for all architectures (x-labels denote the number of hidden units in each layer).

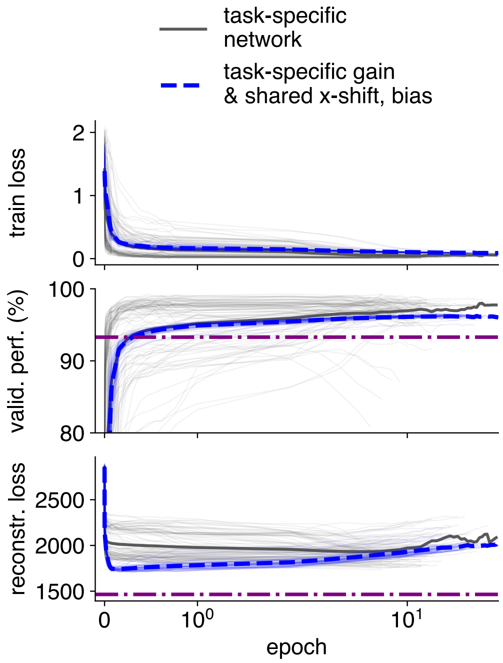

Fig. S2. Train loss (top), validation performance (together with $\triangle$PMD test performance as in Fig 4F for comparison, middle) and reconstruction loss ($\min_C \|\Delta X - C\, W\|$, bottom) during fully supervised learning (as in Fig 3) of the input weight matrix W, for an architecture with one hidden layer of 100 units, and for task-specific networks (grey) and neuron-specific modulations (blue, dashed). The reconstruction loss was evaluated on W during supervised training, and compared with the case were W was given by $\triangle$PMD (purple, dash-dotted), with the aim to investigate whether the supervised approach trained purely on the classification loss also minimizes Eq. (3), so as to elucidate the extent to which supervised training adapts the input weight matrix to the subspace of the data, and how that depends on the task set. To that end, we compare the value of the residual $\min_C \|\Delta X - C\, W\|$ of the reconstruction loss Eq. (3) during training of W in a fully supervised fashion, with the reconstruction loss when W was given by $\triangle$PMD. We assess the case where the entire network was task-specific (feedforward weights included, grey) and the gain-modulated case (blue), and find that the sharp initial reduction in train loss (i.e. the classification loss, top) is associated with a sharp reduction in reconstruction loss (bottom). The reconstruction loss for the gain-modulated network, where a single feedforward weight matrix has to contribute to solving a multitude of tasks, decreased to a value much closer to the $\triangle$PMD optimum than the task-specific network average, where the feedforward weight matrix only has to solve a single task. Thus, supervised training forgets the initialization by adapting feedforward weights to the data subspace, and then fine tunes to the specific set of tasks, as validation performance during the later phase increases from the unsupervised value (obtained with $\triangle$PMD) to its maximum. The unsupervised approach on the other hand is generalist, and admits any task that might be defined post hoc, at the cost of forgoing task-specific fine tuning.

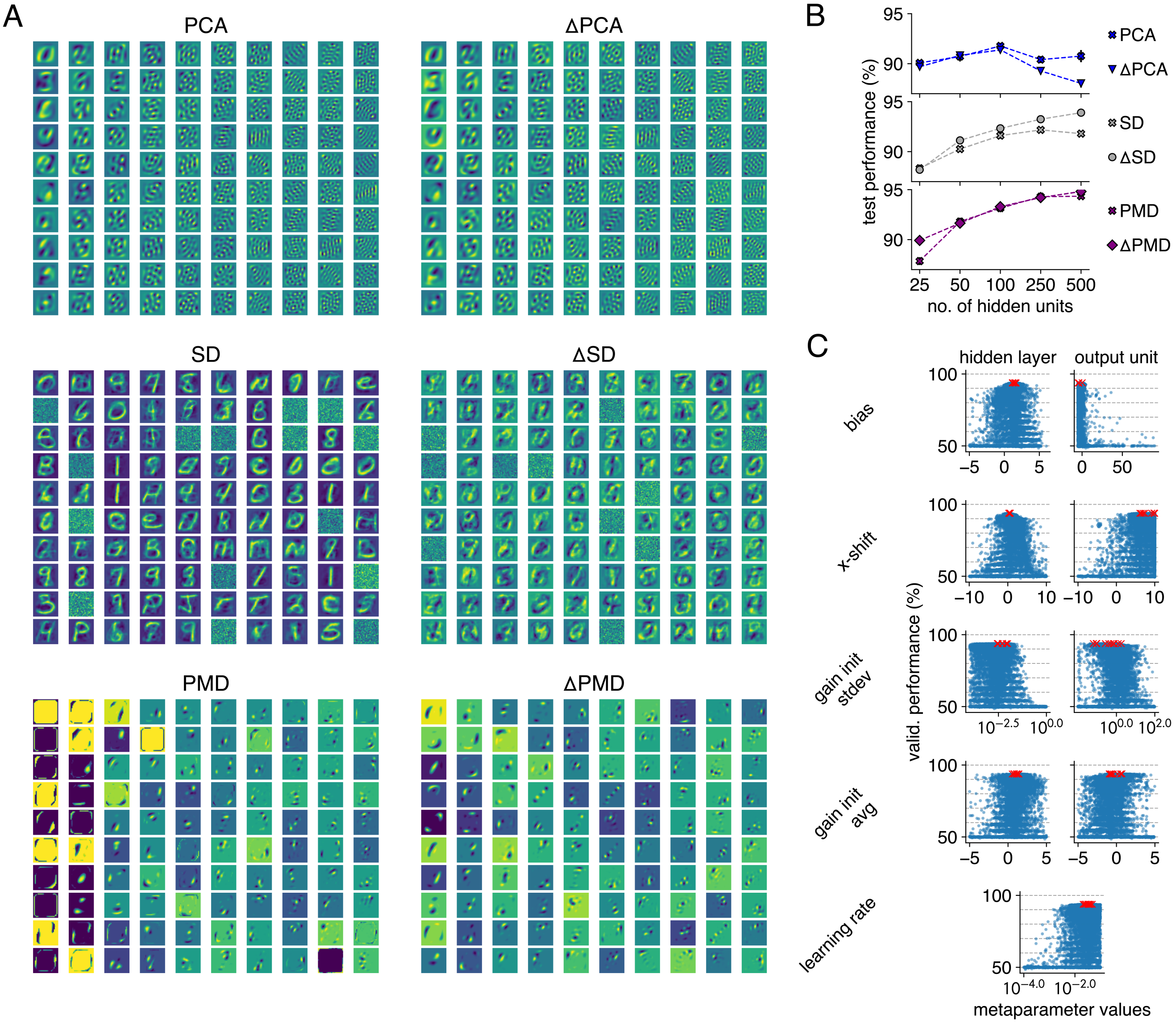

Fig. S3. Supplementary results for the networks with unsupervised feedforward weights and supervised gain adaptation. **A:** Weight vectors to the hidden units ($k = 100$) for the three unsupervised learning algorithms in Fig 4, i.e. principal component analysis (top), applied to data samples (PCA) or difference vectors ($\triangle$PCA), sparse dictionary learning (middle) applied to data samples (SD) or differences ($\triangle$SD), and penalized matrix decomposition (bottom) applied to data samples (PMD) or differences ($\triangle$PMD). **B:** Comparison of performance of gain-modulated networks on multitask EMNIST, between feedforward weights given by applying the respective unsupervised learning algorithms to the data samples versus the difference vectors, as a function of layer size. For PCA and $\triangle$PCA, weight vectors are very similar (A), resulting in similar performance for $k \leq 100$. For larger k, performance decreases as the extra principal components are no longer useful for classification tasks. For SD and PMD applied to data samples vs. differences, the distinct weight vectors (A) result in a performance increase for $\triangle$SD resp. $\triangle$PMD vs. SD resp. PMD. **C:** To combine the unsupervised weights with supervised gain modulation to learn the specific tasks, we performed an evolutionary meta-parameter optimization on the shared bias, the shared x-shift, the gain learning rate and the gain initialization. Note that shared parameters were not only shared across tasks, but also across neurons. Gain initialization was Gaussian with optimized mean (gain init avg) and standard deviation (gain init stdev). Plots show performance of a randomly chosen subset of ten tasks evaluated on the validation set for $\triangle$PMD with $k = 100$, for all configurations tried by the evolutionary algorithm. The ten best metaparameter configurations are marked by red crosses. The optimal metaparameter configuration was determined in this way for each input weight matrix and hidden layer size shown in Fig 4F.

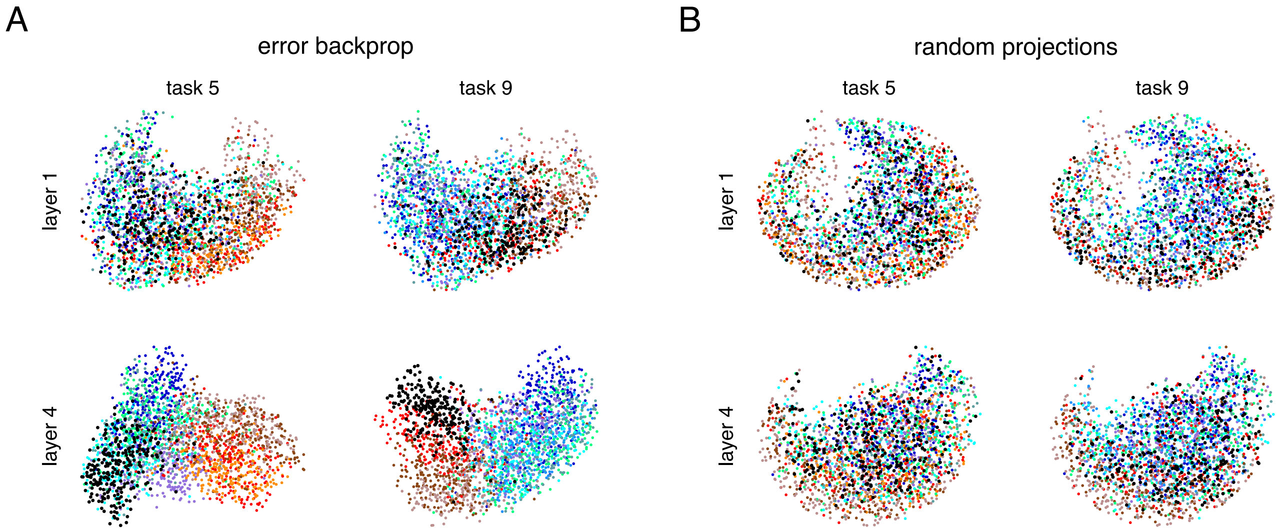

Fig. S4. UMAP of the hidden representations, as in Fig 6F, but for the case of error backprop **(A)** and RP **(B)**.

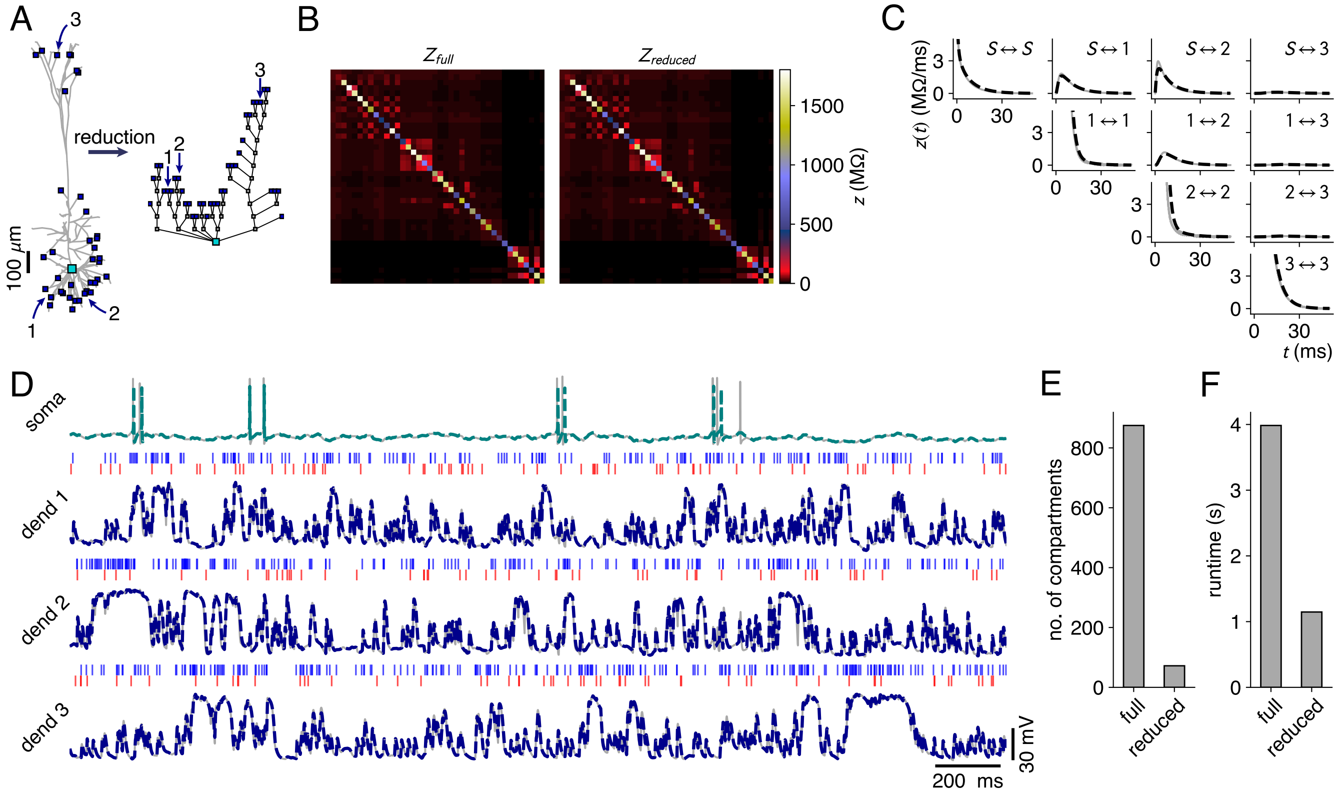

Fig. S5. Validation of the simplification of the biophysical L5 PC model. **A:** Schematic of the reduction. Synaptic input sites (blue) and soma (teal) indicated on the morphology (left) are retained in the simplified model (right). Note that the simplification needs to incorporate all bifurcations between branches with input sites (grey squares on the right) to be accurate (13). **B:** Matrix of steady state input and transfer resistances (diagonal and off-diagonal elements respectively) between the synaptic inputs sites and the soma, computed for the full model (left) and the reduction (right). **C:** Input and transfer resistance kernels (also termed impulse response kernels or the Green's function) between the soma and three randomly selected dendritic sites (as indicated in A). Note that these kernels are symmetric $z_{ij}(t) = z_{ji}(t) \equiv z_{i \leftrightarrow j}(t)$. **D:** Responses in the full model (grey, full line) and the reduction (colored, dashed line) for the soma (teal) and the three dendritic sites (blue). Only the dendritic sites receive synaptic input, in the form of excitatory inputs to AMPA + NMDA synapses (blue) and inhibitory inputs to $GABA_A$ synapses (red). **E:** Number of compartments in the full and reduced models. **F:** Runtime of the full and reduced models on an Apple M1 Max Macbook Pro with 32 GB of RAM for a simulation time of 10000 ms.

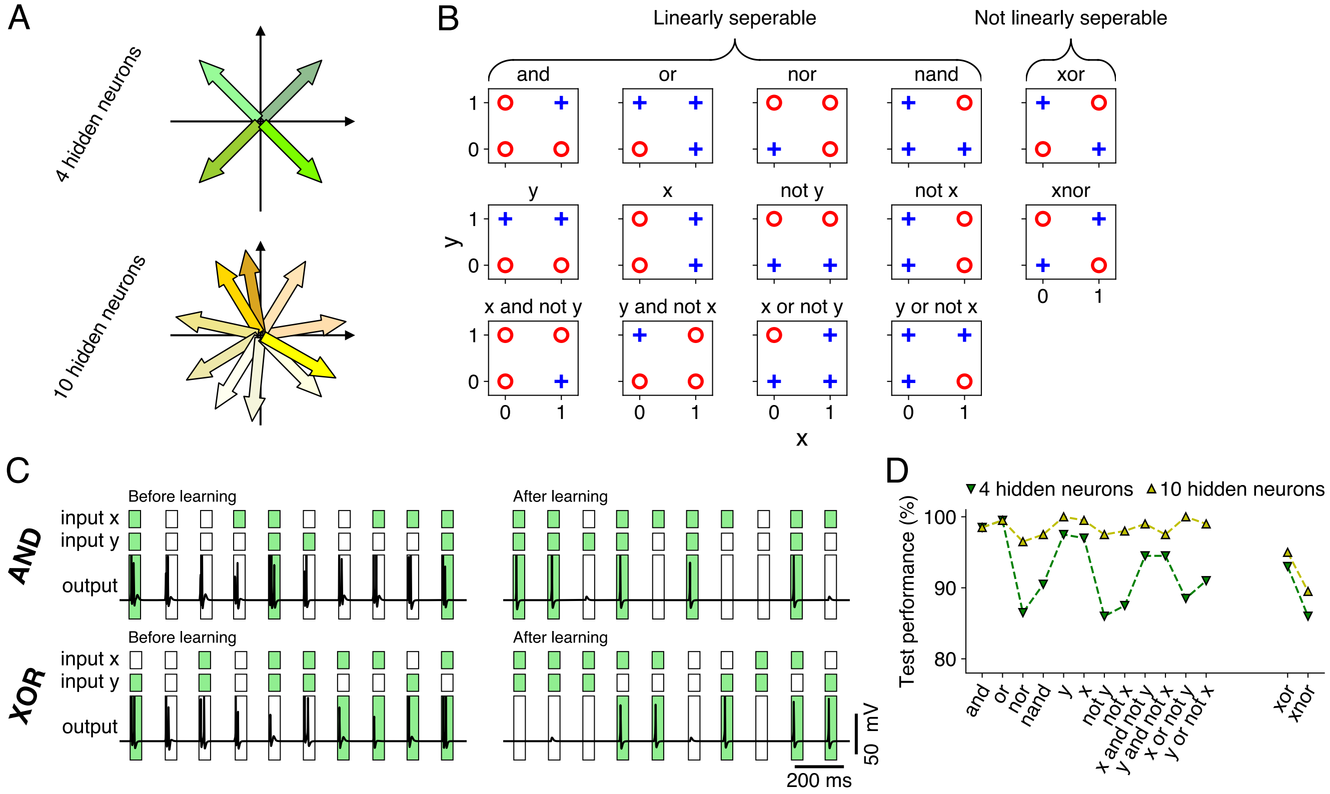

Fig. S6. Application of the biophysically realistic spiking network architecture to all non-trivial boolean functions. **A:** Two network configurations were trained, one with 4 hidden neurons and one with 10 hidden neurons. The weight vectors were distributed so as to allow construction of decision boundaries with normal vectors in any direction of the two-dimensional input space. For 4 hidden neurons (top) we chose the centers of the 4 quadrants, whereas for 10 hidden neurons (bottom) we divided the unit circle in 10 equal parts and sampled a vector at random from each of these parts. **B:** We learn the 14 possible non-trivial boolean classification tasks on inputs $x, y \in \{0, 1\}$. 12 of these tasks are linearly separable (left) and two are linearly non-separable (right). The output neuron of the network should learn to spike in response to the blue crosses, and not spike in response to the red circles. **C:** Voltage response of the output neuron (in the network with 10 hidden neurons) to inputs $x, y \in \{0, 1\}$. Note that input values were sampled in such a way that for each task the network received a balanced set of input combinations with $+$ and $\circ$ targets. Input values $\{0, 1\}$ were converted to a Gaussian burst of 6 ms width containing 20 spikes in case of 1, and no spikes otherwise. The output neuron spikes indiscriminately initially (left) but learns to spike correctly after learning (right), here shown for the "and" (top) "xor" (bottom) task. The apparent variability in spike amplitude is due to a recording time step of 1 ms. **D**: Performance (best of 3 initialization seeds) for the networks with 4 and 10 hidden neurons.

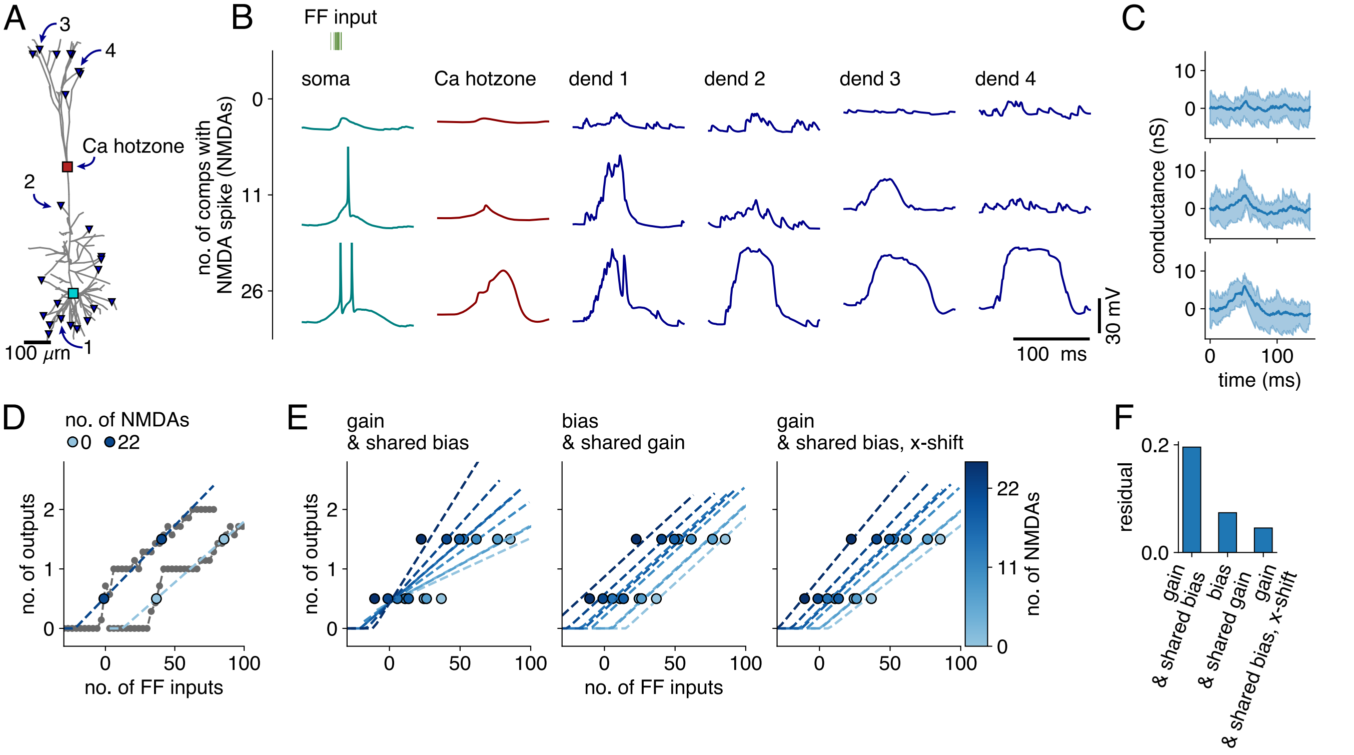

Fig. S7. Contextual modulation of a fully active L5 PC model (1). **A:** Configuration of contextual input sites (blue triangles), the soma (teal square) and a recording site in the apical trunk (near the Ca^{2+} hotzone, red square). Labeled sites (1-4, Ca hotzone) correspond to the plotted traces in B. **B:** Responses to identical feedforward input (top, green) for various levels of contextual input, recorded at the soma (teal), the Ca^{2+} hotzone (red), and selected dendritic compartments (blue, locations as indicated in A). **C:** Effective membrane conductance change for the three modulation levels shown in B, as in Fig 1H. **D-F:** Modeling of the IO relationship as in Fig 2A, B & E. Gain modulation with a shared x-shift and bias is most accurate.

References

1. E Hay, S Hill, F Schürmann, H Markram, I Segev, Models of neocortical layer 5b pyramidal cells capturing a wide range of dendritic and perisomatic active properties. *PLoS computational biology* **7**, e1002107 (2011).
2. NT Carnevale, ML Hines, *The NEURON book.* (2004).
3. G Major, A Polsky, W Denk, J Schiller, DW Tank, Spatiotemporally Graded NMDA Spike/Plateau Potentials in Basal Dendrites of Neocortical Pyramidal Neurons (Supplementary figures). *J. neurophysiology* (2008).
4. P Rhodes, The properties and implications of NMDA spikes in neocortical pyramidal cells. *The J. neuroscience : official journal Soc. for Neurosci.* **26**, 6704–15 (2006).
5. S Rotter, M Diesmann, Exact digital simulation of time-invariant linear systems with applications to neuronal modeling. *Biol. cybernetics* **81**, 381–402 (1999).
6. CE Jahr, CF Stevens, A quantitative description of NMDA receptor-channel kinetic behavior. *The J. neuroscience : official journal Soc. for Neurosci.* **10**, 1830–1837 (1990) ISBN: 0270-6474 (Print)\r0270-6474 (Linking).
7. BF Behabadi, BW Mel, Mechanisms underlying subunit independence in pyramidal neuron dendrites. *Proc. Natl. Acad. Sci. United States Am.* **111**, 498–503 (2014).
8. B Haider, M Häusser, M Carandini, Inhibition dominates sensory responses in the awake cortex. *Nature* **493**, 97–100 (2013) ISBN: 1476-4687 (Electronic)\r0028-0836 (Linking).
9. A Paszke, et al., PyTorch: An Imperative Style, High-Performance Deep Learning Library in *Advances in Neural Information Processing Systems 32.* pp. 8024–8035 (2019).
10. DP Kingma, J Ba, Adam: A Method for Stochastic Optimization in *Proceedings of the 3rd International Conference for Learning Representations.* (arXiv), (2015) arXiv:1412.6980 [cs].
11. F Pedregosa, et al., Scikit-learn: Machine Learning in Python. *J. Mach. Learn. Res.* **12**, 2825–2830 (2011).
12. FA Fortin, FM De Rainville, MA Gardner, M Parizeau, C Gagné, DEAP: Evolutionary Algorithms Made Easy. *J. Mach. Learn. Res.* **13**, 2171–2175 (2012).
13. WA Wybo, et al., Data-driven reduction of dendritic morphologies with preserved dendro-somatic responses. *eLife* **10**, e60936 (2021) Publisher: eLife Sciences Publications, Ltd.
14. A Krizhevsky, Learning Multiple Layers of Features from Tiny Images, (University of Toronto, Toronto), Technical Report TR-2009 (2009).
15. A Coates, H Lee, AY Ng, An Analysis of Single-Layer Networks in Unsupervised Feature Learning. *Proc. 14th Int. Conf. on Artif. Intell. Stat.* **15**, 215–223 (2011).
16. B Illing, J Ventura, G Bellec, W Gerstner, Local plasticity rules can learn deep representations using self-supervised contrastive predictions in *Advances in Neural Information Processing Systems.* (Curran Associates, Inc.), Vol. 34, pp. 30365–30379 (2021).
17. S Ioffe, C Szegedy, Batch Normalization: Accelerating Deep Network Training by Reducing Internal Covariate Shift in *Proceedings of the 32nd International Conference on Machine Learning.* (JMLR.org, Lille), (2015) arXiv:1502.03167 [cs].
18. T Chen, S Kornblith, M Norouzi, G Hinton, A Simple Framework for Contrastive Learning of Visual Representations in *Proceedings of the 37th International Conference on Machine Learning.* (PMLR), pp. 1597–1607 (2020) ISSN: 2640-3498.
19. K He, X Zhang, S Ren, J Sun, Delving deep into rectifiers: Surpassing human-level performance on imagenet classification. *Proc. IEEE Int. Conf. on Comput. Vis.* **2015 Inter**, 1026–1034 (2015) arXiv: 1502.01852v1 ISBN: 9781467383912.
